# MRTF specifies a muscle-like contractile module in Porifera

**DOI:** 10.1038/s41467-022-31756-9

**Published:** 2022-07-15

**Authors:** J. Colgren, S. A. Nichols

**Affiliations:** 1 Department of Biological Sciences, University of Denver, Denver, USA; 2 Present Address: Sars International Center for Marine Molecular Biology, Bergen, Norway

**Keywords:** Evolutionary developmental biology, Musculoskeletal development, Calcium signalling, Contractile proteins

## Abstract

Muscle-based movement is a hallmark of animal biology, but the evolutionary origins of myocytes are unknown. Although believed to lack muscles, sponges (Porifera) are capable of coordinated whole-body contractions that purge debris from internal water canals. This behavior has been observed for decades, but their contractile tissues remain uncharacterized with respect to their ultrastructure, regulation, and development. We examine the sponge *Ephydatia muelleri* and find tissue-wide organization of a contractile module composed of actin, striated-muscle myosin II, and transgelin, and that contractions are regulated by the release of internal Ca^2+^ stores upstream of the myosin-light-chain-kinase (MLCK) pathway. The development of this contractile module appears to involve myocardin-related transcription factor (MRTF) as part of an environmentally inducible transcriptional complex that also functions in muscle development, plasticity, and regeneration. As an actin-regulated force-sensor, MRTF-activity offers a mechanism for how the contractile tissues that line water canals can dynamically remodel in response to flow and can re-form normally from stem-cells in the absence of the intrinsic spatial cues typical of animal embryogenesis. We conclude that the contractile module of sponge tissues shares elements of homology with contractile tissues in other animals, including muscles, indicating descent from a common, multifunctional tissue in the animal stem-lineage.

## Abstract (web summary)

Myocytes are a key cell type that enable animal movement, but their evolutionary origins remain unclear. Colgren and Nichols describe molecular and functional similarities between a contractile module in tissues of a sponge and muscle tissues in other animals, indicating a common evolutionary origin.

## Introduction

Animal movement is predominantly enabled by the coordinated activity of two cell types: myocytes (contractile cells of the muscular system) and neurons (signaling cells of the nervous system). But not all animals have these cell types—sponges lack both yet are still capable of coordinated tissue movements during whole-body contractions. Understanding how sponges contract has key relevance for understanding the evolutionary origins of animal movement.

**Fig. 1:**
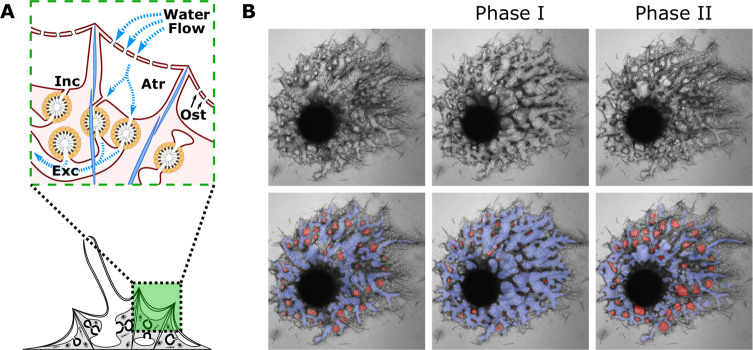
Sponge body plan and contractions. **A** The tent-like outer layer is supported by spicules (light blue) and is composed of two epithelia, which house a thin extracellular matrix (pink) containing migratory cells. The incurrent system (Inc) comprises the atrium and incurrent canals. Water is drawn into the atrium through incurrent pores (ostia; Ost) and into choanocyte chambers (orange), then into excurrent system (Exc) and through the osculum where it exits the sponge. **B** Still images from a mechanically induced contraction show that a ~40 min contraction cycle involves at least two phases. During phase I (*t* = 15 min), incurrent canals (red) narrow as excurrent canals (blue) widen. During phase II (*t* = 36 min), incurrent canals widen as excurrent canals narrow.

Strictly sessile, sponges must actively pump water through an internal canal system for feeding, gas exchange, waste removal, and sexual reproduction. The canal system is partitioned into incurrent cavities (atria) and canals, feeding chambers lined by flagellated cells (choanocytes) that generate direction flow and phagocytose bacteria, and excurrent canals that channel wastewater to exhalant openings (oscula) (Fig. 1A). Contractions serve to clear canal blockages and maintain water flow. At the initiation of a contraction, incurrent pores close and canals narrow, and internal water pressure increases to dislodge debris^1^ (Fig. 1A).

Lab-grown freshwater sponges are useful models for studying contractions because they are small, transparent, and amenable to microscopy. In *Ephydatia muelleri*, contraction cycles can be described as biphasic. During phase I, atrial volume decreases, and incurrent canals narrow as water is displaced into excurrent canals which expand in diameter. During phase II, atrial volume and incurrent canal diameter increase as excurrent canals narrow (Fig. 1B). This is a progressive sequence, leading from the incurrent tissues of the system to the excurrent tissues, and propagates towards oscula^1^.

From a signaling perspective, it is known that canal blockage (natural or induced by the addition of Sumi ink) activates ciliated sensory cells, which release nitric oxide (NO). Nitric oxide diffuses across tissues and is modulated by glutamate and gamma-aminobutric acid (GAGA), initiating contraction^2–4^. A major gap in this model is that the cellular mechanisms of the contractile response remain essentially uncharacterized. How is the contractile force generated structurally, and how are contractions regulated at the level of cellular physiology? Answers to these questions are needed to clarify whether sponge contractile tissues are similar to non-muscle contractile tissues in other animals such as epithelia that undergo apical constriction, or to muscles?

**Fig. 2:**
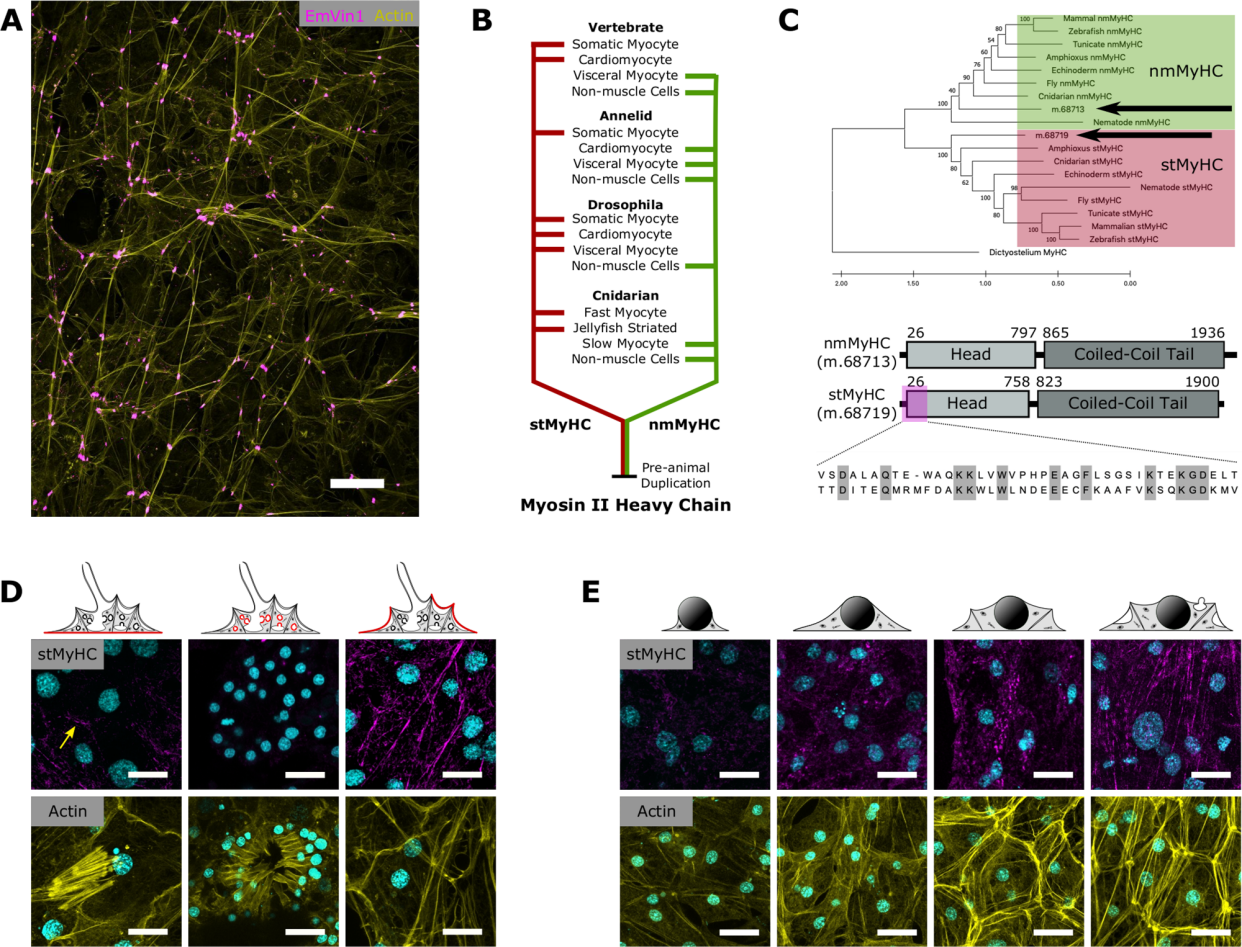
Organization of actomyosin bundles. **A** Organization of actin-bundles (yellow) in incurrent tissues, with cell-junctions marked by vinculin staining (magenta)^71^. **B** Duplication and divergence of the Myosin II Heavy Chain. **C** The type II MyHC proteins found in *E. muelleri* (labeled with arrows) fall into either the stMyHC clade or nmMyHC clade. Phylogeny determined by the maximum likelihood method. Tree rooted with MyHC from *Dictyostelium*. Support values represent 1000 bootstrap iterations. Bottom panels show the basic structure of two type II MyHCs found in *E. muelleri* and the alignment of epitope region used to generate stMyHC antibody. **D** stMyHC immunostaining in the basopinacoderm (left), a choanocyte chamber (middle), and incurrent tissues (right). Samples were stained for DNA (magenta), stMyHC (cyan; top), and actin (yellow; bottom). Yellow arrow shows cell boundary staining in basopinacoderm. Immunostaings were performed on multiple sponges over three independent experiments with consistent results. **E** Developmental series of actin-bundles in incurrent pinacocytes stained for DNA (magenta), stMyHC (cyan; top), and actin (yellow; bottom). Immunostaining and phalloidin stainings were performed on multiple sponges over two independent experiments with consistent results. Scale bars 25 μm in **A** and 10 μm in **D**, **E**.

In well-studied bilaterian animal models with muscle tissues, fast-contracting somatic myocytes are involved in voluntary or reactive movements, and slow-contracting visceral myocytes are involved in organ movements and tissue tension^5^. In both, contractions require interactions between actin filaments and type-II myosin, composed of two myosin heavy chains (MyHCs), two regulatory light chains (RLCs), and two essential light chains. The contraction speed of different cell and tissue types reflects the kinetics of the MyHC expressed. For example, striated-muscle myosin heavy chain (stMyHC) is found in fast-contracting skeletal and cardiac muscles of vertebrates, and smooth and striated invertebrate muscles^6–8^ (Fig. 2B). Non-muscle myosin heavy chain (nmMyHC) is found in slow-contracting vertebrate smooth muscle, and some invertebrate visceral muscles, but also in non-muscle contractile contexts such as cell motility, cytokinesis, and apical constriction^9^.

Bilaterian myocytes can be further distinguished by their regulatory mechanisms (see below). In fast contracting myocytes, the tropomyosin/troponin C complex hinders stMyHC from binding to actin but is released by Ca^2+^^10^. Troponin C-regulation is unique to bilaterian muscle. A potentially more ancient mechanism is the MLCK pathway, in which cytoplasmic Ca^2+^ binds to calmodulin, activating MLCK to phosphorylate the RLC of myosin II^11,12^. The MLCK pathway functions in muscle and non-muscle contractile contexts, and appears to regulate non-bilaterian muscles^7,13^.

During development, unique transcription factor combinations specify the identity of either cardiac and smooth muscles, or striated muscles in vertebrates (see below)^5^. These are well-conserved between species and indicate cell type homology between lineages^14^. An interaction central to the developmental specification of all muscle types is between a myocardin-related transcription factor (MRTF) and a MADS-box transcription factor—either serum response factor (SRF) or myocyte enhancer factor 2 (Mef2). Vertebrate smooth/cardiac muscle development also involves transcription factors from GATA, NK homeobox, and Fox families, and vertebrate striated/skeletal muscle development also involves E12 and MyoD transcription factors (restricted to bilaterians)7.

Here, we examine tissues of the sponge *Ephydatia muelleri* and find evidence of a contractile module with evolutionary links to muscle. Specifically, contractions depend upon the motor-activity of striated-muscle myosin II and are regulated by MLCK pathway, and the development of the contractile module appears to involve MRTF as part of a possible transcriptional complex that includes SRF and Fox-family transcription factors.

## Results

### Contractile actin-bundles in pinacocytes contain stMyHC

The tissues lining the atrial cavity and canals are believed to play a primary role in the contraction in sponges. These tissues are regionally differentiated but are all composed of endothelial-like cells (pinacocytes) that form a watertight barrier, express genes involved in contraction^15,16^, and decrease in area during contraction^17^. Moreover, in *E. muelleri*, pinacocytes contain linear actin-bundles that align between adjacent cells, exhibiting tissue-wide organization (Fig. 2A).

Searching the transcriptome^18^ we identified two type II myosin heavy chains, which clearly fall into stMyHC and nmMyHC groups (Fig. 2C). We generated and validated a custom antibody targeting a divergent region in the motor head for stMyHC (Fig. 2D, Supplementary Fig. 9). To test whether these actin-bundles are associated with myosin II, we immunostained for stMyHC (Fig. 2E). Staining was initially diffuse but increased throughout development. In fully differentiated tissues, stMyHC was organized into linear structures that resemble actin-bundle orientation (Fig. 2F), but co-staining was incompatible with fixation conditions. To further test for association with actin-bundles we treated sponges with latrunculin B, an inhibitor of actin polymerization, and observed that loss of stMyHC staining mirrored the dynamics of actin-bundle disassembly (Supplementary Fig. 1).

### Contractions are regulated by the MLCK pathway

**Fig. 3:**
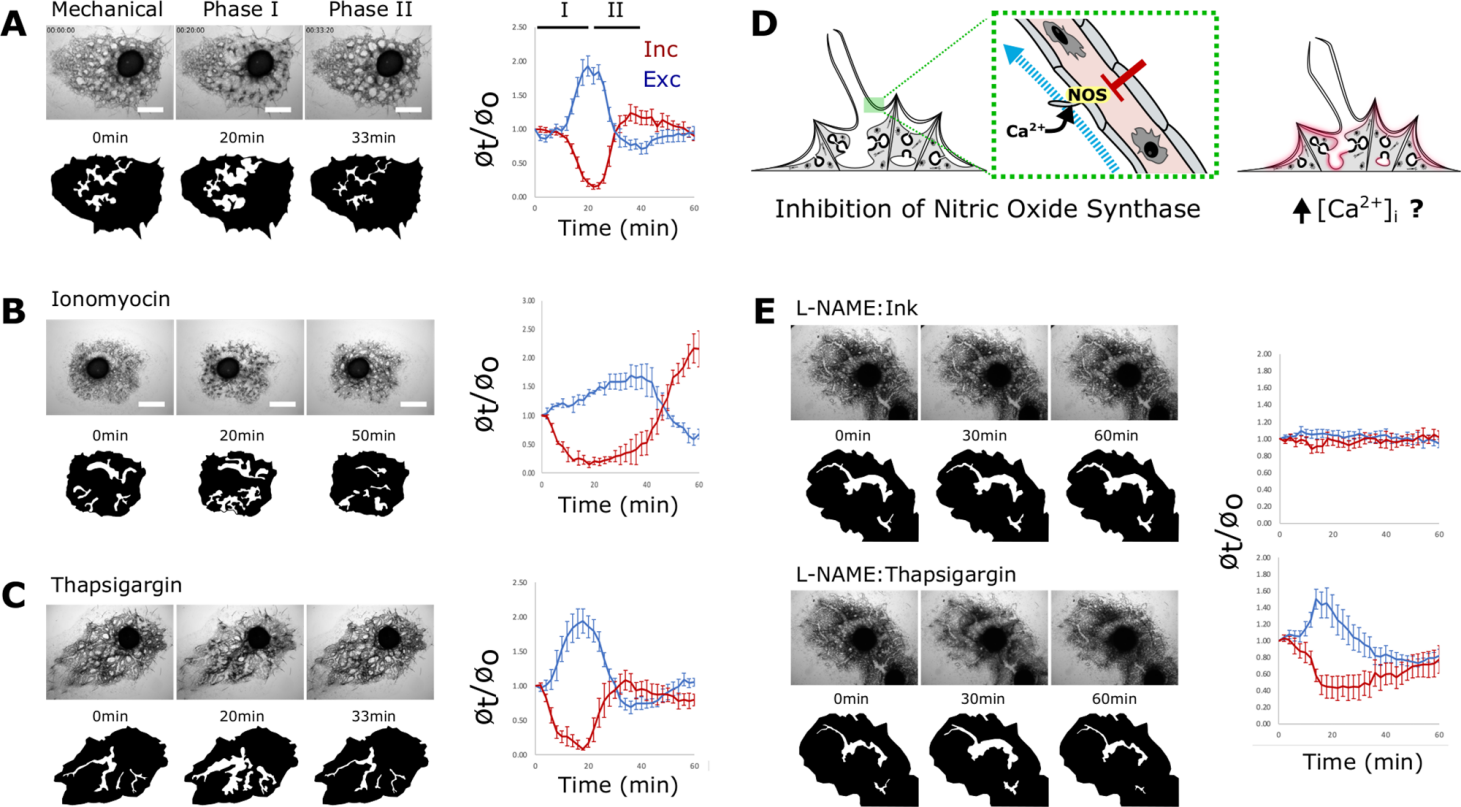
Contractions are regulated by calcium. **A**–**C** Time-lapse images depicting sponge contractions **A** stimulated mechanically, **B** by 300 nM Ionomycin, or **C** by treatment with 1 nM thapsigargin. Below each image, an excurrent canal is highlighted in white to show how its diameter changed over the treatment time-course. Graphs (right) plot phase I and II contraction dynamics (*n* = 5 or 6 individual measurements for incurrent and excurrent canals per sample). **D** Simplified workflow, as sensory cells are known to require external calcium2 does raising intracellular calcium concentration downstream of them still elicit a contraction? **E** Top: time points of a sponge treated with 20 µM L-NAME and challenged to contract with 1:1000 Sumi Ink. Bottom: the same sponge treated with 1 nM thapsigargin. Graphs show the average canal diameter relative to the first frame for both incurrent (red) and excurrent (blue) canals (*n* = 6 individual measurements for incurrent and excurrent canals per sample). Analysis was also performed on sponges from independent experiments and showed consistent canal dynamics. Data are presented as mean values +/− SEM. Scale bars 500 μm.

Previous studies have shown that sponge contractions abate in Ca^2+^/Mg^2+^-free medium (CMFM)^1,19^. To test the inverse of this, whether elevated cytoplasmic Ca^2+^ can induce contractions, we treated *E. muelleri* in CMFM with the calcium ionophore, ionomycin, and with the SERCA pump inhibitor, thapsigargin. Both treatments induced a strong biphasic contraction cycle (Fig. 3A). In ionomycin-treated sponges, contractions appeared to be more drawn-out and less intense compared with thapsigargin-treated sponges, which closely mirrored mechanically induced contractions at the tested concentrations. We interpret this as a difference in the kinetics of the molecules, as well as the mechanism of action; thapsigargin likely requires sufficient intracellular stores to elicit a response.

Since treatments were administered globally, the observed response could be a secondary effect downstream of NO signaling from activated sensory cells. To decouple sensation and contraction, we treated sponges with the NO-synthase inhibitor L-NAME (Fig. 3B). Sponges deficient for NO signaling did not contract in response to treatment with Sumi ink (which activates sensory cells) but exhibited a strong response to thapsigargin (Fig. 3C). This suggests that thapsigargin is acting on cells downstream of the sensory cells, which is consistent with the presence of endoplasmic reticulum-associated Ca^2+^ stores in contractile tissues.

**Fig. 4:**
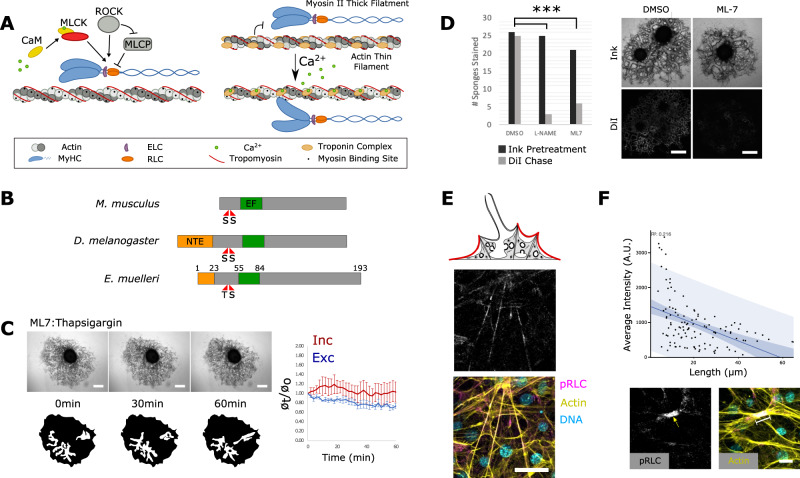
Contractions require MLCK activity. **A** Fast-contracting myocytes are regulated by the Troponin C/Tropomyosin complex, whereas slow-contracting myocytes are regulated by the MLCK pathway. **B** Predicted domain structure of *E. muelleri* RLC; a 23 amino acid N-terminal extension (NTE) exists based on the location of phosphorylatable residues and EF-hand domain. **C** 1 nM thapsigargin had limited effect on sponges pretreated with 1 µM ML-7 to inhibit MLCK activity (*n* = 6 individual measurements) Data are presented as mean values +/− SEM. **D** Sponges were treated with Sumi Ink to block water flow, then treated with DiI to test for restored flow as an indicator of contractile activity. The ratio of sponges that stained positive for DiI was significantly lower following treatment with 50 μg/mL L-NAME and 1 µM ML-7 compared with DMSO treated controls (χ2(df2, *N* = 70) = 13.08, *P*-value = 0.001). Contraction assays were performed three independent times with consistent results. **E** pRLC (grayscale and magenta) immunostaining of the apical pinacoderm, counterstained with phalloidin (yellow) and Hoechst (cyan). **F** Scatter-plot generated by overlaying pRLC and F-actin images and measuring pixel intensity in the pRLC channel along the length of the actin-bundle between two adhesion plaques. Simple regression analysis was performed (*n* = 118, *r*^2^ = 0.218) dark blue represents 90% confidence band and light blue represents 90% prediction band. Below are example images with raw pRLC channel and a merged image showing a brightly pRLC-stained, short actin-bundle. Immunostainings for pRLC were performed on multiple sponges over three independent experiments with consistent results. Scale bars 500 μm in **C**, **D**, 10 μm in **E**, and 5 μm in **F**.

With respect to the regulation of Ca^2+^ dependent contraction (Fig. 4A), *E. muelleri* has homologs of calmodulin, MLCK, and an RLC ortholog with conserved functional residues (Fig. 4B). If the release of internal Ca^2+^ is upstream of MLCK signaling, then contractions should be disrupted by the MLCK inhibitor ML-7. To test this, we first established a concentration of Sumi ink that permanently blocked water flow in L-NAME treated sponges (i.e., sponges deficient for NO synthesis), but could be cleared by contraction of untreated sponges. Ink clearance and the reestablishment of flow was determined by post-treating sponges with DiI to see if it entered canals. We found that ML-7-treated sponges were unable to clear ink and restore flow, nor could they be induced to contract with thapsigargin (Fig. 4C, D). Whereas ML-7 targets MLCK and is predicted to affect only a subset of myosin II activity, treatment with more general myosin ATPase inhibitors is expected to have global effects on myosin activity. We found that blebbistatin and para-amino blebbistatin caused a complex phenotype similar to phase I of the contraction cycle, but sponges never entered phase II and could not be induced to do so with thapsigargin (Supplementary Fig. 2).

In vertebrates, the RLC is the substrate for MLCK during smooth-muscle contraction^20^. To test for RLC phosphorylation during contraction, we immunostained sponges for phosphorylated RLC (pRLC) and saw staining of contractile bundles at the sponge surface (Fig. 4E). Their length was negatively correlated (*R*^2^ = 0.216, *n* = 118) with pRLC staining, indicating that increased phosphorylation of the RLC is associated with their contraction (Fig. 4F).

Collectively, these results support that contractions depend upon the release of ER-associated Ca2+ stores, downstream of NO signaling. Elevated Ca2+ then activates MLCK, leading to increased phosphorylation levels of RLC associated with contractile actin-bundles in pinacoderm tissues.

### MRTF drives development of the contractile module

**Fig. 5:**
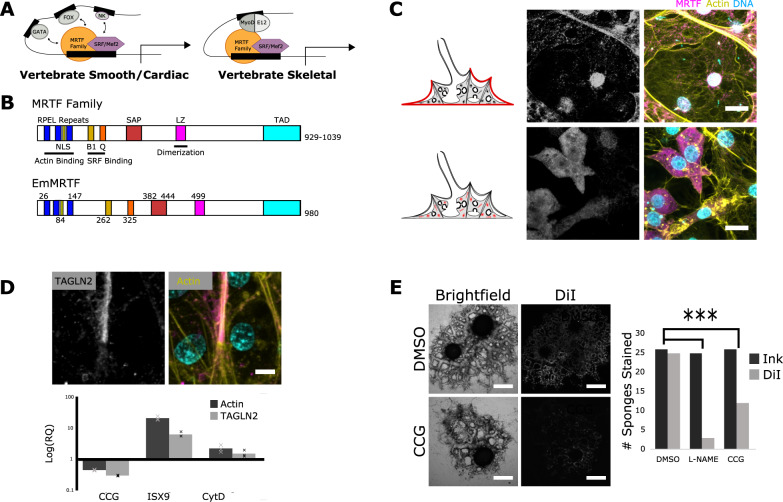
Development of contractile structures depends on MRTF-activity. **A** Myocytes are developmentally specified by a transcriptional complex that includes MRTF interactions with SRF or Mef2. **B** Predicted domain organization of *E. muelleri* MRTF compared with vertebrate homologs (NLS; nuclear localization signal, B1, basic rich domain; SAP, SAP domain; LZ, leucine zipper; TAD, transcription activation domain). **C** Confocal images of pinacocytes (top) and archeocytes (bottom) immunostained for MRTF (grayscale and magenta), phalloidin (yellow), and Hoechst (cyan). Immunostainings for MRTF were performed on multiple sponges over five independent experiments with consistent results. **D** Top: confocal images of an actin-bundle immunostained for TAGLN2 (grayscale and magenta), phalloidin (yellow) and Hoechst (cyan). Bottom: qPCR of TAGLN2 in response to treatment with CCG-203971 (20 μM), ISX (50 μM), and cytochalasin D (10 μM) (*n* = 3 technical replicates and 3 biological replicates). Data are presented as mean values (bars) with individual experimental values overlaid (crosses). Immunostainings for TAGLN2 were performed on multiple sponges over four independent experiments with consistent results. **E** Sponges pretreated with CCG-203971 were unable to clear ink-blockages by contraction (χ2(df2, *N* = 77) = 12.64, *P*-value = 0.002). A redrawn image from *The evolutionary origin of bilaterian smooth and striated myocytes* by Brunet T, et al., modified to generalize protein families and highlight central interaction, expressing relationships based on our phylogenetic analysis of MRTF family proteins, Creative Commons—Attribution 4.0 International—CC BY 4.0. Scale bars 500 μm (**E**), 5 μm in **C**, and 2 μm in **D**. Contraction assays were performed three independent times with consistent results.

To test for conserved developmental mechanisms involved in specifying contractile cell-fate in sponges and bilaterians, we focused on the transcriptional cofactor, MRTF (Fig. 5A). In bilaterians, MRTF homologs are broadly expressed, but inhibited through the interaction between G-actin with N-terminal RPEL (RPxxEL) repeats^21^. Actin polymerization disrupts this interaction, exposing a nuclear localization signal and leading to nuclear translocation^22,23^. In the nucleus, MRTF interacts with SRF or Mef2 to drive the expression of contractile genes and can induce the differentiation of myocytes^24–27^ and myofibroblasts^28^, and is involved in the regulation of epithelial-mesenchyme transitions^29,30^. Loss of function studies in mice have shown that the loss of myocardin results in early embryonic lethality due to improper formation of vascular smooth muscle^31^. Loss of MRTF-B also results in early embryonic lethality due to cardiac defects^32,33^. Though most ubiquitously expressed, loss of MRTF-A is not lethal but results in deficiencies in lactation, likely resulting from loss of myoepithelial tissue^34^.

*E. muelleri* has a single, broadly expressed (Supplementary Fig. 3) MRTF ortholog that is predicted to have conserved RPEL repeats, suggesting that its activity may also be regulated by interactions with G-actin (Fig. 5B). Using a custom antibody, we found that this protein was predominantly cytoplasmic in undifferentiated archeocytes (stem cells), and nuclear in pinacocytes (Fig. 5C and Supplementary Fig. 4), where it presumably acts as a transcriptional co-factor. Known targets of MRTF regulation in bilaterians include the transgelin family (e.g., calponin, SM22alpha, MP20), which can be muscle-specific^21,35^. *E. muelleri* has three transgelin paralogs. Of these, we found that transgelin 2 (EmTAGLN2) was specifically localized to contractile bundles in pinacocytes (Fig. 5D and Supplementary Fig. 5).

Because MRTF activity is regulated by G-actin, it is possible to pharmacologically manipulate this interaction. Highly specific MRTF inhibitors such as CCG-203971^36,37^ have been identified, as well as activators that act through less specific mechanisms. One of these, *N*-cyclopropyl-5-(thiophen-2-yl)isoxazole-3-carboxamide (ISX) is an MRTF activator^38–40^ that drives cardiomyocyte differentiation in vivo^38^. ISX also drives the expression of secretory programs and neuronal differentiation through the transcription factor NeuroD1 in a Mef-2-dependent manner^41–43^. However, as sponges lack neurons and NeuroD1, we reasoned that it may act primarily on MRTF in sponges. To test this, we monitored EmTAGLN2 (a predicted MRTF-target) expression in response to CCG-207319 and ISX treatment and observed corollary changes; CCG-207319 treatment caused a decrease in EmTAGLN2 expression, whereas ISX treatment caused an increase in EmTAGLN2 expression (Fig. 5D). Since ISX is less specific than CCG-207319, we also corroborated this result by treatment with cytochalasin D—a potent MRTF-activator through competitive binding of G-actin^44^—and similarly observed an increase in EmTAGLN2 expression. From a functional perspective, MRTF-inhibited sponges had a reduced ability to clear Sumi ink—an indicator of diminished contractile activity (Fig. 5E).

**Fig. 6:**
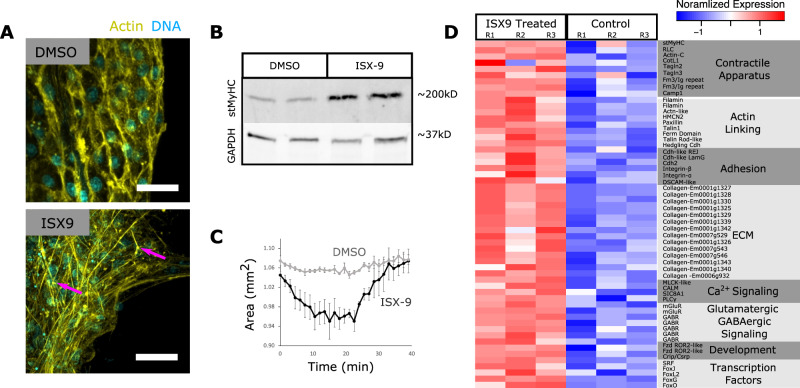
MRTF drives contractile tissue differentiation in primmorphs. **A** Primmorphs treated with 50 µM ISX or DMSO and stained with phalloidin (yellow) and Hoechst (cyan). **B** Western Blot showing elevated stMyHC levels in ISX-treated samples. Raw and uncropped scans in source data file. **C** Contraction dynamics of ISX-treated primmorphs in response to thapsigargin. Data are presented as mean values +/− SEM. (*n* = 3 biological replicates). **D** Heatmap of select transcripts that were differentially expressed in ISX-treated samples. Primmorphs treated with ISX and vehicle controls were maintained in an attachment-free environment in nine independent experiments. Phalloidin stainings were performed twice, total protein extraction was performed on one sample, and total RNA was extracted from three independent experiments with consistent results. Scale bars 10 μm.

To test the effects of MRTF-activation on contractile tissue differentiation, we dissociated juvenile sponges and treated archeocyte-enriched cell fractions with either DMSO or ISX and placed them in an attachment-free environment (this allows for the formation of primary aggregates—primmorphs—but delays differentiation). After three days, control primmorphs lacked contractile-bundles, whereas primmorphs treated with ISX developed linear actin-bundles that aligned at adhesion plaques (Fig. 6A). These stained positive for pRLC (Supplementary Fig. 6) and treated primmorphs contained elevated levels of stMyHC (Fig. 6B). Thapsigargin had no effect on control primmorphs but induced contractions in ISX-treated samples, which exhibited a 15.0 (+/−5.5)% reduction in cross-sectional area, followed by return to resting size (Fig. 6C).

### MRTF activates myogenic factors, signaling, contractile, and adhesion genes

To understand the transcriptional response of primmorphs to MRTF activation, we sequenced mRNA from ISX or DMSO treated primmorphs. Differential expression analysis of 16,712 mapped transcripts revealed that 1390 were upregulated and 1091 were downregulated (Supplementary Figs. 7–8). We interpret upregulated genes as candidate targets of MRTF regulation, and downregulated genes as archeocyte-enriched. The ISX-treated samples had elevated expression of genes involved in contraction, signaling, development, and adhesion (Fig. 6D). Contraction-related genes included stMyHC (LogFC = 0.65, *P* = 0.038), which was validated by western blot (Fig. 6B), TAGLN2 (LogFC = 1.03, *P* = 1.0E^−3^), and components of the Ca^2+^-dependent MLCK pathway including MLCK-like serine/threonine kinase (LogFC = 1.58, *P* = 6.29E-5), calmodulin (LogFC = 1.55, *P* = 0.002), sodium/calcium exchanger 1 (SLC8A1) (LogFC = 1.05, *P* = 0.029), and Phospholipase C gamma (PLCγ) (LogFC = 1.01, *P* = 0.005) (Fig. 6D). Upregulated signaling genes included metabotropic glutamate receptors and GABA receptor subunits (Fig. 6D), consistent with a role for glutamatergic and GABAergic signaling in contractile behavior^2,3^. Upregulated developmental factors included the myogenic transcription factor SRF (LogFC = 1.18, *P* = 0.001). Four Forkhead transcription factors showed increased expression, including Fox-L2 (LogFC = 1.07, *P* = 0.008), FoxG (LogFC = 1.31, *P* = 4.16E^−5^), which is expressed in myocytes of invertebrates^45^, FoxO, and FoxJ1 (LogFC = 0.60, *P* = 0.016 and LogFC = 1.86, *P* = 0.001 respectively). The phylogenetically broad muscle marker Crip/Csrp^46^ was also upregulated (LogFC = 1.10, *P* = 0.001).

Some of the most highly upregulated genes belonged to the collagen family, suggesting a role for pinacocytes in the secretion of extracellular matrix (Fig. 6D). As many as fourteen collagens had elevated expression in ISX-treated samples. Adhesion molecules, including cadherins, integrins, and down syndrome cell adhesion molecule, had increased expression levels as well (Fig. 6D). Though many upregulated genes correspond to the transcriptional profile of pinacocytes based on scRNA-seq data^47^, upregulation of silicatein—a sclerocyte marker—suggests that ISX treatment caused differentiation of other cell types as well, directly or indirectly.

## Discussion

A challenge for tracing the ancestry of myocytes is that muscles are very diverse in modern animals. For example, like vertebrates, the ascidian *Ciona robusta* and the annelid *Platynereis dumerilii* have striated myocytes that express stMyHC, are regulated by the troponin C/tropomyosin complex, and are developmentally specified by a skeletal muscle-like transcriptional complex. They also have smooth muscles that express nmMyHC and are patterned by a smooth/cardiac muscle-like transcriptional complex^5,48^. However, in a divergence from the vertebrate paradigm, the flatworm *Schistosoma mansoni* has smooth muscles that express stMyHC and are patterned by a skeletal-muscle like complex that includes MyoD^49,50^, but contractions are regulated by MLCK phosphorylation of the RLC. Among non-bilaterian animals, cnidarians have smooth and striated myocytes with epithelial-like organization that express either stMyHC or nmMyHC and are regulated by the MLCK pathway (where known). Their developmental specification is not yet characterized, but they lack the skeletal muscle transcription factor MyoD^7^. Also, ctenophore myocytes predominantly have smooth ultrastructure (with one exception^51^), express stMyHC, but lack troponin C and MyoD^7,52^.

Here, we establish that *E. muelleri* also contains a contractile module with elements of homology to contractile tissues in other animals, including myocytes. Actomyosin bundles containing stMyHC and transgelin exhibit tissue-wide organization in pinacoderm tissues that line internal body cavities and canals. Contraction depends upon the release of ER-associated Ca^2+^ stores and MLCK regulation of stMyHC, and the development of the module appears to depend upon MRTF-activity. Our interpretation of this contractile module as muscle-related is corroborated by single-cell sequencing data from the related species, *Spongilla lacustris*, which indicate that pinacocytes, and another cell type—myopeptidocytes—cluster with myocytes from other animals (myopeptidocytes are solitary cells found between tissues that express contractile genes including nmMyHC)^45,47^. Single-cell RNA sequencing of the demosponge *Amphimedon queenslandica* also revealed co-expression of key components of the actin-based contractile apparatus in pinacocytes^15^.

Although sponge contractile tissues and muscles have similarities that can only be explained by common ancestry, we do not assert that this reflects their one-to-one homology. This is partly due to the similarity of muscle and non-muscle contractile mechanisms, but also because invertebrate muscles are often multifunctional. For example, the epitheliomuscles of *Hydra* function in contraction, the formation of an epithelial barrier, innate immunity, and regeneration^53^. In the planarian *Schmidtea mediterranea*, muscle also acts as a connective tissue that secretes ECM proteins (including 19 collagens) and signaling molecules that provide spatial cues for regeneration from neoblasts^54^. High expression of numerous collagens has also been found in the epitheliomuscles of *Nematostella vectensis* (30 in the circular body muscles), which is interpreted as a sign of multifunctionality^55^. Similarly, sponge pinacoderm tissues form an endothelial-like barrier to the environment, are capable of phagocytosis^56^, and express genes involved in sensation, metabolism, and defense^47^. Also, in an intriguing parallel with *S. mediterranea* and *N. vectensis*, MRTF-induction of contractile tissue development was associated with the upregulation of as many as 14 collagen genes.

Our findings can inform hypotheses about the organization of contractile tissues in the first animals, as well as the sequence of events that gave rise to early muscles. Specifically, the epithelial-like nature of contractile tissues in both sponges and cnidarians suggests that this represents the ancestral state, and that narrowly specialized myocytes emerged later. This fits well with the view of epithelial tissues as the “building blocks” of animal body plans^57^, which may have been among the first tissues to evolve. It has been hypothesized that myoepithelia represent a retained feature of an ancient muscle precursor^17,58^. The first animals were undoubtedly aquatic, so myoepithelia may have functioned to maintain tissue tension in the context of a hydrostatic skeleton or may have functioned in peristalsis-like behaviors like those seen in burrowing of the anemone *Nematostella* vectensis, or canal constriction in *E. muelleri*.

Depending on the phylogenetic position of sponges, which is contentious^59,60^, the long-held view that they lack muscles has been interpreted as evidence that myocytes evolved after sponges diverged from other animals, or that myocytes were lost in sponges^61^. Our results clarify that the contractile module of muscle tissues predates modern animals. Consistent with this interpretation, the stMyHC, and nmMyHC paralogs diverged in the holozoan stem lineage^7,62^, and the choanoflagellate *Choanoeca flexa* forms colonies that resemble a polarized epithelium with coordinated contractile behaviors^63^.

Finally, the possible role of MRTF in specifying contractile tissues in *E. muelleri* helps to explain the plasticity and regenerative capacity of sponges. Evidently, without the need for intrinsic spatial cues characteristic of embryogenesis, sponges can develop from archeocyte-enriched aggregates and gemmules, and adult tissues can remodel in response to flow dynamics^64–67^. In vertebrates, MRTF is an actin-regulated force-sensor involved in muscle plasticity and regeneration^22,68,69^, and our data indicate that similar mechanisms are operating in *E. muelleri*. This supports a model in which environmental-feedback mechanisms drive contractile tissue development, and we speculate that this could be common in sponges. Even during sexual reproduction, there is limited correlation between embryonic patterning and adult tissue identity^70^. It is conceptually plausible that the evolution of animal developmental mechanisms involved a transition in which ancient environmental feedback mechanisms were later harnessed by genetically encoded, intrinsic patterning mechanisms.

## Methods

### Sponge collection and cultivation

Gemmules of *E. muelleri* were collected from an unnamed lake in the Brainard Lake Recreation Area in the Colorado Rocky Mountains (40°04'48.0“N 105°32'34.6“W) and stored in autoclaved lake water (LW) at 4 °C. Before use, gemmules were treated with 1% hydrogen peroxide for 5 min, washed thoroughly with autoclaved lake water, and plated in either six-well plates (CellTreat #229106) or coverslip-bottom dishes (MatTek Corporation #P35G-1.5-10-C) in LW containing 100 µg/mL ampicillin. Plates/dishes were then placed in a dark cabinet at room temperature (RT) until hatching (~3 days). After hatching, water was renewed daily without the addition of ampicillin.

### Induction of contraction by mechanical agitation, Sumi ink, and elevated Ca2+

Sponges were induced to contract using mechanical agitation by placing the plate or dish on a vigorous rocking platform for 3 min before transfer to an inverted microscope for imaging. Alternatively, contractions were induced with Sumi Ink (Yasutoma #KY6) by incubating sponges in 1:1000 solution for 10 min, followed by LW washes. To test for Ca^2+^ dependence of contractions, sponges were placed in 2 mL LW and left on the microscope stage for 1 h to confirm they were not contracting prior to treatment. After 5 min of imaging, treatments were applied by gently removing 1 mL of LW and replacing it with 1 mL of either thapsigargin (Tocris #1138) or ionomycin (Sigma-Aldrich #I0643) at twice the desired final concentrations of 1 or 30 nM, respectively. To induce contractions in the absence of NO signaling or external Ca^2+^, sponges were pretreated with either 50 µg/mL L-NAME (Cayman Chemical #80210), 0.1 mM EDTA, or deionized water (Milli-Q, Millepore). Time lapse images were collected, generally, at a rate of one image every 20 s and stacks were compiled into videos using ImageJ software. Major excurrent canals were manually annotated from still images. For changes in canal diameter, at least six incurrent and excurrent canal areas were chosen for each sponge and diameter across the same plane was measured every 6th frame over the course of the video. The ratio of diameter at time *t* to the initial diameter at *t* = 0 was then taken to represent change in diameter over the course of treatment. Graphs of canal dynamics were generated using Microsoft Excel (version 16)™.

### Antibody production and validation

Polyclonal antibodies against stMyHC and MRTF were produced in rabbits (Syd Labs), and polyclonal antibodies against TAGNL2 and TAGNL3 were produced in chickens (Pacific Immunology). The coding region of each antigen (Supplemental Sequences) was cloned into the pET His6 GST TEV LIC cloning vector (Addgene, plasmid #29655) from synthesized gBlocks (Integrated DNA technologies) (stMyHC) or from the *E. muelleri* cDNA library. Recombinant proteins were expressed in *Escherichia coli* (Rosetta strain DE3; EMD Millipore) and purified with Pierce Glutathione resin (ThermoFisher Scientific #16101) following the manufacturer’s protocol. Antibodies were affinity purified on a column made by coupling each recombinant protein to AminoLink Resin (ThermoFisher Scientific #20381) and validated by Western blotting and by Peptide Competition Assay (Supplementary Figs. 9–12).

### Immunostaining

Sponges were grown in glass-bottom dishes for 3–4 days post hatching and then fixed using one of two methods: (1) to prepare samples for pRLC (Cell Signaling Technology #3671 T), MRTF, TAGNL2, TAGNL3, and vinculin^71^ staining, sponges were fixed in 3.7% formaldehyde in EtOH for 50 min at room temperature; (2) to prepare sponges for stMyHC staining, sponges were fixed in Carnoy’s solution (60% EtOH, 30% chloroform, 10% glacial acetic acid) for 3 min at RT, followed by a gentle 10 min wash in 100% EtOH at RT. All samples were then washed in PBST and blocked with PBST containing 3% BSA for 1 h at RT. Primary antibodies in blocking solution were applied for 1 h at RT, or overnight at 4 °C (1:100 for anti-pRLC, 1:200 for anti-MRTF, 1:250 for anti-TAGLN2, 1:500 for anti-TAGLN3, 1:1000 for anti-EmVin1 and 1:500 for anti-stMyHC). Secondary antibodies, either anti-rabbit AF488 (Life Technologies) or anti-Chicken IgY AF 647 (Invitrogen #A32933), were applied at a 1:1000 dilution, together with 1:120 phalloidin AF 568 (ThermoFIsher Scientific #A12380), and 1:100 Hoechst dye in blocking solution. Samples were incubated for 45 min at RT in the dark, washed in PBST, and mounted in mounting medium (0.1% Propyl gallate, PBS pH 7.6, 90% glycerol). Samples were imaged on an Olympus Fluoview FV3000 confocal microscope.

In order to understand the developmental dynamics of stMyHC, sponges were fixed in 8 h time-intervals after attachment to the dish. The only change to the fixation protocol described above was that the glacial acetic acid component of Carnoy’s solution was added dropwise during the first minute to help preserve the delicate structure of the newly formed tissues. Since actin filaments were not preserved using this fixation method, we instead performed a timed sequence of latrunculin B treatments to test whether stMyHC staining was disrupted in conjunction with actin-bundle dissolution. Specifically, gemmules were plated in glass-bottom dishes and grown until canals and choanocytes were clearly visible. Sponges were then treated with 20 μM latrunculin B in LW for either 1, 5, 10, or 30 min, then fixed and stained using appropriate methods for either stMyHC or F-actin.

To test for elevated pRLC staining in contracted tissues, 10 z-stacks were taken through comparable regions of the pinacoderm using the same confocal settings for relaxed and contracted samples. Projections of the images were made in Fiji^72^ and the F-actin and pRLC images were merged. pRLC staining intensity was measured as pixel intensity and expressed as a ratio to actin-bundle length (nine biological and three technical replicates). Measurements were obtained from nine biological and three technical replicates, and a simple regression analysis was performed for pixel intensity and actin-bundle length in RStudio^73^.

### HCR RNA fluorescent in situ hybridization of MRTF and SRF

Multiplexed HCR RNA-FISH was performed with custom split initiator probes ordered from Molecular Instruments®. Sponges were grown in coverslip-bottom dishes (Mattek) for 6 days. Cellbrite-fix 640 (Biotium) was added at 1:1000 dilution for 15 min, and sponges were then washed once in lakewater. Immediately after washing, samples were fixed in ice-cold 4% (v/v) paraformaldehyde in ¼ Holtfreter’s solution overnight at 4 C and then washed with ¼ Holtfreter’s. A dehydration series was performed to transfer the samples to 100% MeOH and then transferred to 100% EtOH before rehydration into 1× PBST. Samples were digested with 5 μg/mL protease K for 90 s and digestion was halted with 2 mg/mL glycine. Samples were then postfixed in 4% (v/v) paraformaldehyde in 1× PBST for 1 h at room temperature and were washed into 2× SSC. Hybridization and amplification were performed using buffers supplied by Molecular Instruments® and following the general protocol with minor modifications. Samples were prehybridized for 30 min in hybridization buffer at 37 C. Samples were then hybridized with 0.5, 2, 5, 10, and 10 pmol probes for each target for ~16 h at 37 C in humidified chambers, on a rocking platform. Samples were then washed into 2× SSC and brought to room temperature. Samples were pre-amplified in provided amplification buffer for 30 min at room temperature and 24 pmol of amplification hairpins for each target were prepared by snap cooling, separately, in amplification buffer. These were combined and added to the sample for 16 h at room temperature in the dark. Samples were then washed into 1× PBST and stained with 1:100 Hoechst dye before mounting.

### Testing the role of MLCK and MRTF in physiological contractions

Gemmules were plated in 24-well dishes and treated with the 1 μM ML-7 (MLCK inhibitor^74,75^), or 50 μg/mL L-NAME for 2 h, or 20 μM or 50 μM CCG-203791 (MRTF inhibitor^76,77^) for 3 days beginning at tent stage, with treatment refreshed daily. To disrupt flow, sponges were each treated with 1:20 (v/v) Sumi ink for 10 min, carefully washed with LW, and allowed to sit, undisturbed, for 3 h (long enough to allow ink blockages to be cleared in sponges capable of contraction). To test if water flow was restored by contraction, samples were then treated with 1:1000 (v/v) DiI (ThermoFisher Scientific #D282) in LW for 10 min in the dark and washed with LW, which under normal flow conditions strongly stains choanocyte chambers. Samples were manually screened and imaged via epifluorescence microscopy for DiI staining within water canals. Laser-level and exposure time was set based on control sponges and DiI staining was judged based on overlap with Sumi ink-stained choanocyte chambers. Washout experiments and extended treatments were performed to confirm sponges remain viable under treatment conditions.

### qRT-PCR

Gemmules were plated in six-well format on 22 mm coverslips with ~10 sponges per well. For MRTF inhibition, sponges were treated with 20 μM CCG-203971^76,77^ in LW, beginning at ‘tent-stage’ (i.e., before choanocyte chambers formed) and with treatment refreshed daily. For MRTF activation, sponges were treated with 50 μM ISX9^38,39^ beginning at tent-stage and with treatment refreshed daily, or with 10 μM cytochalasin D (ThermoFisher Scientific #PHZ1063) for 30 min prior to harvesting (cytochalasin D disrupts the MRTF/g-actin association through competitive binding of g-actin^78^). RNA was extracted by washing sponges off of coverslips with Trizol Reagent (ThermoFisher Scientific #15596-018) in a 50 mL tube, followed by ethanol precipitation and rehydration in nuclease-free water. The concentration of purified total RNA was measured by spectrophotometry and an equal mass from each extraction was used to generate cDNA using Takara RNA to cDNA EcoDry mix (Takara #639549). qRT-PCR was performed using a BioRad iQ5 Real-Time PCR detection system (BioRad #170-9780), with an initial denaturing step at 95 °C for 30 s followed by a two-step cycle; 95 °C for 10 s, 60 °C for 55 s, repeated 45 times. A melt-curve was performed after the run to verify the signal was not due to the formation of primer-dimers. Prior to performing the qRT-PCR reaction, a dilution series of purified cDNA library template was used to calculate the amplification efficiency for each of the primer sets in order to verify that it was >1.8 per cycle (2.0 equates to 100% efficiency or doubling each cycle).

### Activation of MRTF in archeocytes

Approximately 50 gemmules were grown to 3 days post-hatching, then dissociated in LW containing 1 mM EDTA and passed through a 70 µm cell strainer. Cell suspensions were centrifuged at 1000 × *g* for 5 min to enrich for archeocytes. The supernatant was removed by pipetting and the cell pellet was resuspended in LW containing 50 µM ISX or DMSO. Using a wide-bore pipette tip, 25 µL of the cell suspension was plated as a hanging drop and maintained for 2–3 days.

Contraction assays were performed by transferring aggregates to the center of a coverslip-bottom dish and adding thapsigargin to a final concentration of 1 nM. Aggregates were photographed every 30 s for 60 min to create time-lapse videos. Time-lapse series were analyzed in Fiji to measure the changes to aggregate size in response to thapsigargin. Immunostaining of aggregates was conducted as described above, except aggregates were stained in 1.5 mL tubes instead of in dishes.

### RNAseq and differential gene expression analysis

Approximately 100 gemmules were grown till 3 days post-hatching, then dissociated as described above. Cells were resuspended in treatment solution (LW, 50 μM ISX) or in control solution (LW, DMSO), and were maintained in 10 mL of solution in a 60 mm petri dish on an orbital rocker to prevent attachment. After 24 h, primmorphs were collected and total RNA was extracted using Trizol Reagent. Experiments were performed in triplicate. Generation of a poly-A-selected library and paired-end sequencing on Illumina HiSeq 4000 platform was performed by Novogene (Sacramento California). Adapter sequences were removed from the raw reads and were quality filtered using fastp^79^. Cleaned reads were mapped to the *E. muelleri* genome^80^ using HISAT2^81^, and transcripts were assembled using StringTie^82^ and htseq-count^83^. Differential expression analysis was performed using EdgeR^84,85^ and was visualized from normalized expression using HeatMap2^86^. Gene ontology analysis was performed using BLAST2GO Pro^87^. The identity of proteins of interest was assessed by performing reciprocal BLAST^88^, searching Pfam^89^, and by phylogenetic analysis using phylogeny.fr^90^. Phylogenetic trees and analysis of proteins of interest in this study are available in the supplement (Supplementary Figs. 13–15).

### Reporting summary

Further information on research design is available in the Nature Research Reporting Summary linked to this article.

## Supplementary information

Supplementary Information

Description of Additional Supplementary Files

Supplementary Movie 1

Supplementary Movie 2

Supplementary Movie 3

Supplementary Movie 4

Supplementary Movie 5

Supplementary Data 1

Reporting Summary

## Data Availability

Sequences and accession numbers for all genes and proteins analyzed in this study are made available in the supplement. Raw RNAseq reads have been deposited at the NCBI SRA (accession number/BioProject PRJNA718521, BioSamples SAMN18537458, SAMN18537459, SAMN18537460, SAMN18537461, SAMN18537462, SAMN18537463). Raw confocal image files are available at figshare (10.6084/m9.figshare.20003945). Source data are provided with this paper.

## Source data

Source Data
