# Supplementary material for: MRTF specifies a muscle-like contractile module in Porifera
Source: Nat Commun. 2022 Jul 15;13:4134. doi: 10.1038/s41467-022-31756-9 (PMC9287330; doi:10.1038/s41467-022-31756-9)
Supplement: Supplementary file 1 — Supplementary Information [file 41467_2022_31756_MOESM1_ESM.pdf]

SUPPLEMENTARY INFORMATION

Supplementary Figures

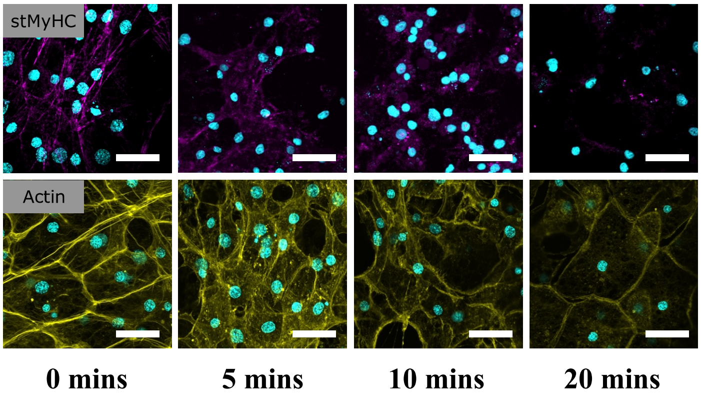

Supplementary Figure 1. Loss of stMyHC signal mirrors actin bundles following treatment with Latrunculin-B. Juvenile sponges were treated with 20µM Latrunculin B for increasing amounts of time prior to fixation. stMyHC signal (cyan; top panel) can be seen to become more diffuse and less organized between 5 and 10 minutes following treatment. This is consistent with the loss of the actin bundles of the incurrent pinacocytes visualized by phalloidin staining (yellow; bottom panel). DNA shown in magenta. Latrunculin-B treatment was performed in two independent experiments with consistent results. Scale bars 20µm.

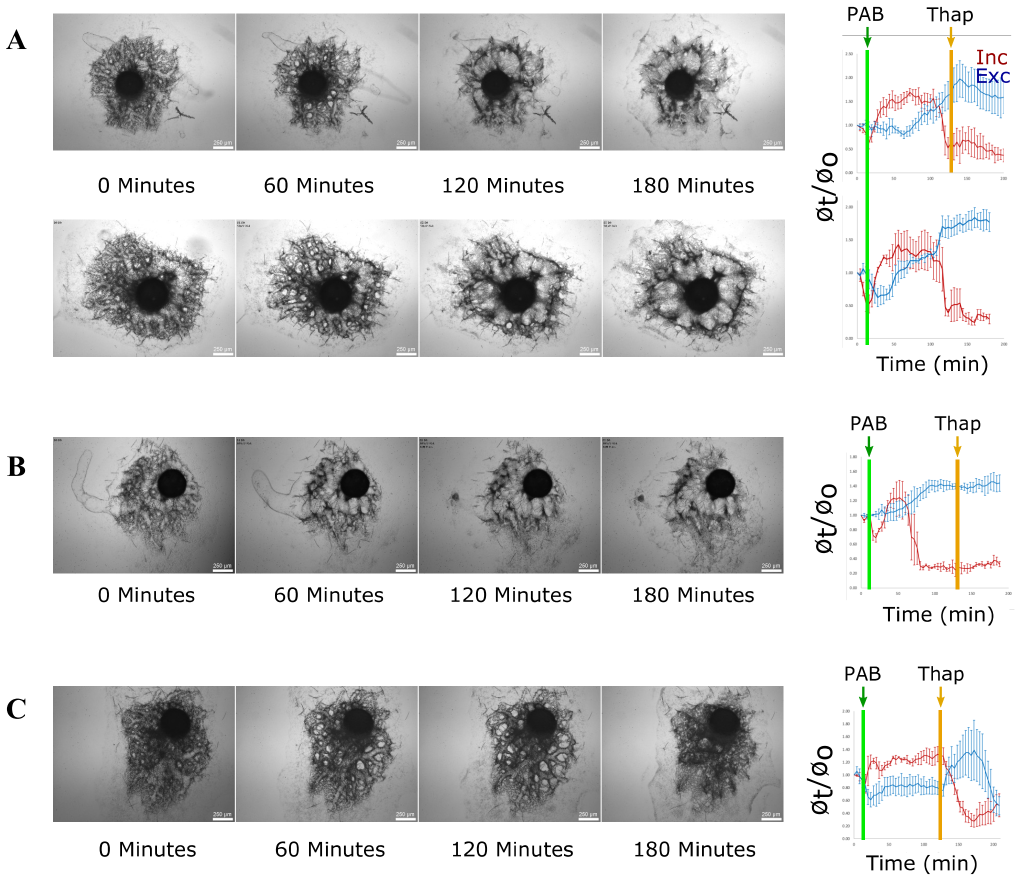

Supplementary Figure 2. Treatment with para-amino blebbistatin. (**A**) Treatment with 50µM PAB for 2 hours causes narrowing of incurrent canals and widening of excurrent canals, similar to phase I of a contraction cycle. Subsequent application of thapsigargin had limited effect. Washout experiments indicate the 4 hour exposure to 50 µM PAB is not toxic. (n=3 independent measurements for incurrent and excurrent canals per sample) (**B**) Treatment with 100 µM PAB for 2 hours had a similar effect, but washout experiments indicate that 4 hour exposure to 100 µM PAB is toxic. (n=3 independent measurements for incurrent and excurrent canals) (**C**) Treatment with 25 µM PAB for 2 hours had no evident effect on canal dynamics, and thapsigargin induced a normal contraction (n=3 independent measurements for incurrent and excurrent canals). Each treatment with thapsigargin stimulation was performed twice in independent experiments with consistent results. Data are presented as mean values +/- SEM.

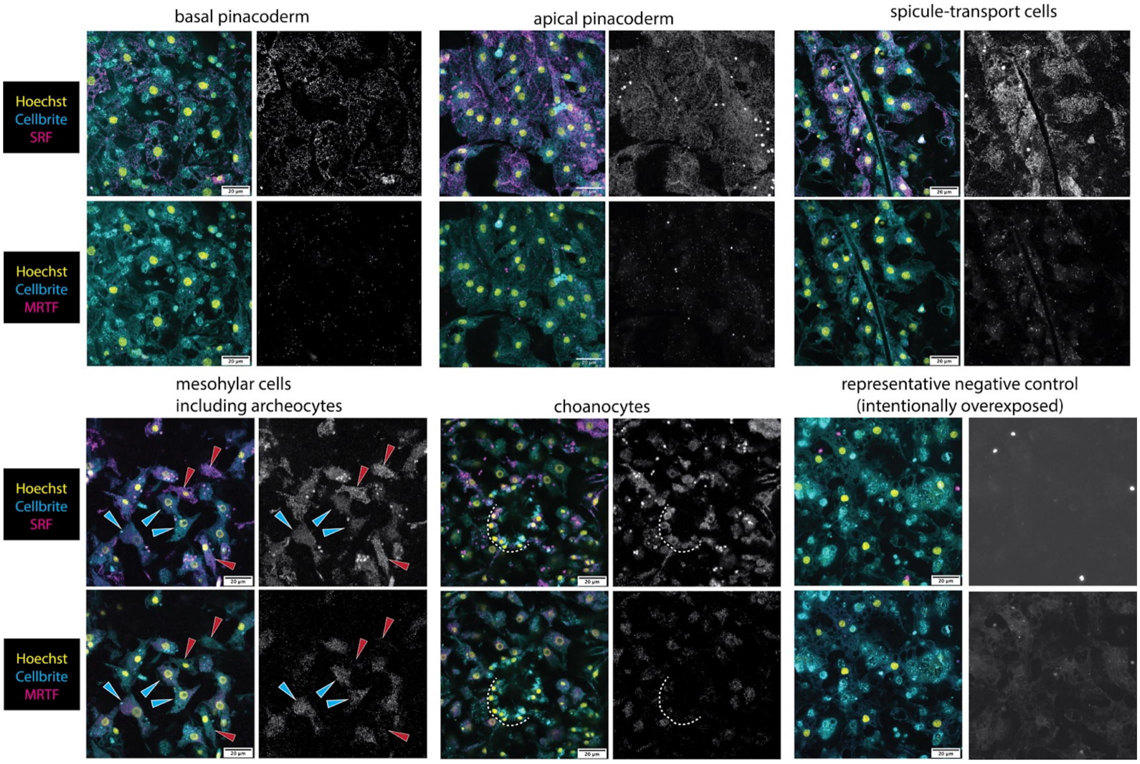

Supplementary Figure 3. HCR-fluorescent *in situ* hybridization of SRF and MRTF in Ephydatia. As in scRNA seq data from *Spongilla lacustris* (closely related FW species), SRF and MRTF were found to be broadly expressed in *Ephydatia*, with SRF expressed at much higher levels. Probes were multiplexed so that expression levels could be monitored at the same time, in the same cells, but channels were separated for the purposes of presentation. Briefly, SRF was detected in the basopinacocytes, apical pinacocytes, spicule transport cells, and many migratory cell types in the mesohyl (with lowest levels in archeocytes), and choanocytes. MRTF levels were quite low but above the background levels of the negative control samples (amplifier-only controls); contrast was increased to enhance visibility. MRTF is clearly present in archeocytes. (Red arrows = various mesohylar cells, blue arrows = archeocytes).

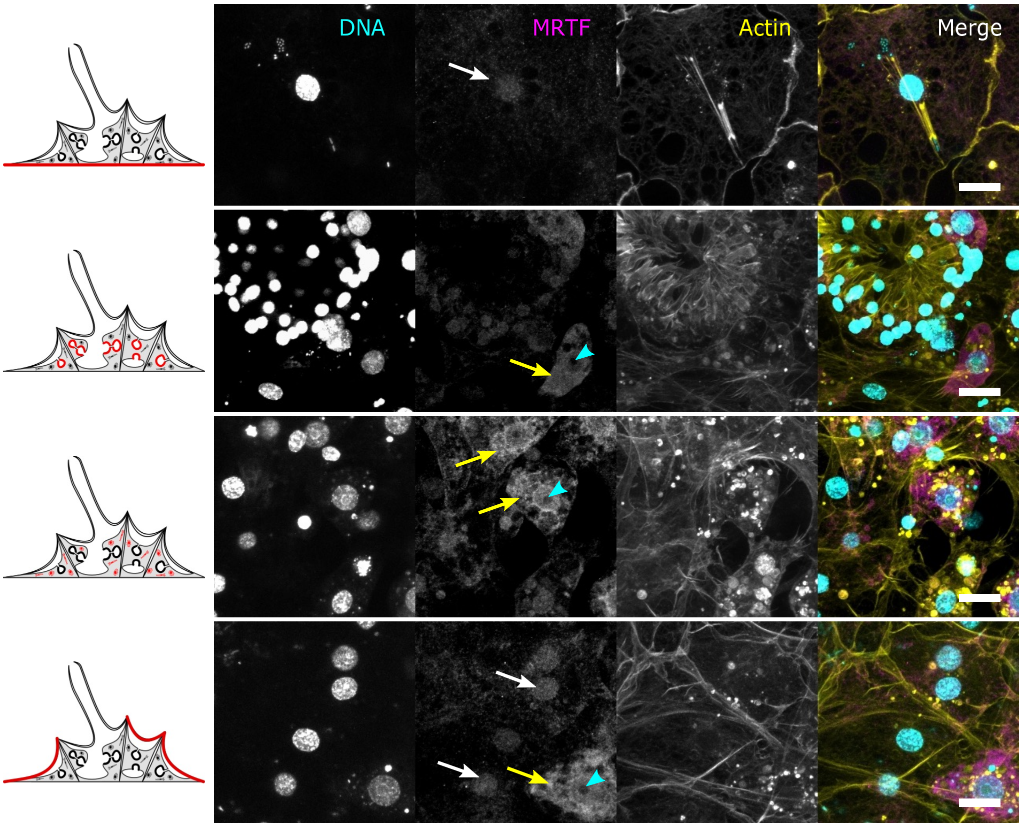

Supplementary Figure 4. MRTF staining in various regions of juvenile *E. muelleri.* Maximum intensity projections of confocal stacks through the basopinacoderm (top), choanoderm (second), mesohyl (third) and pinacocytes of the incurrent tissue system (bottom). From left to right, raw images of DNA, MRTF, and actin, followed by merged images (DNA, cyan; MRTF, magenta; actin, yellow). White arrows highlight MRTF signal in the nucleus while yellow arrows highlight MRTF signal in the cytoplasm. Cyan arrows point to prominent nucleolus found in archeocytes. Immunostainings for MRTF were performed on multiple sponges over five independent experiments with consistent results. Scale bars 10µm.

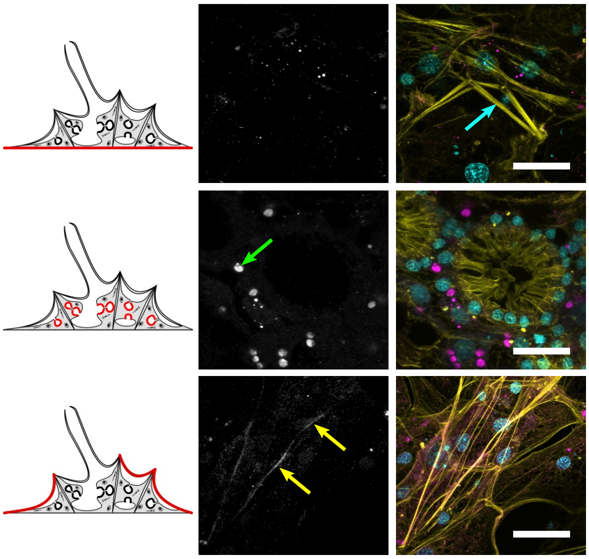

Supplementary Figure 5. Staining for EmTAGLN2 in actin bundles of the incurrent tissue system. Maximum intensity projections of confocal stacks through the basopinacoderm (top), choanoderm (middle), and apical pinacoderm (bottom). Raw antibody channel shown in gray next to merge images showing DNA (cyan), actin (yellow), and EmTAGLN2 (magenta). Cyan arrow shows large stress fiber-like structure in a basopinacocytes, which does not stain for EmTAGLN2. Green arrow highlights auto-fluorescent algae symbiont, and yellow arrow highlights EmTAGLN2 staining along linear filaments. Immunostainings for TAGLN2 were performed on multiple sponges over four independent experiments with consistent results. Scale bars, 10µm.

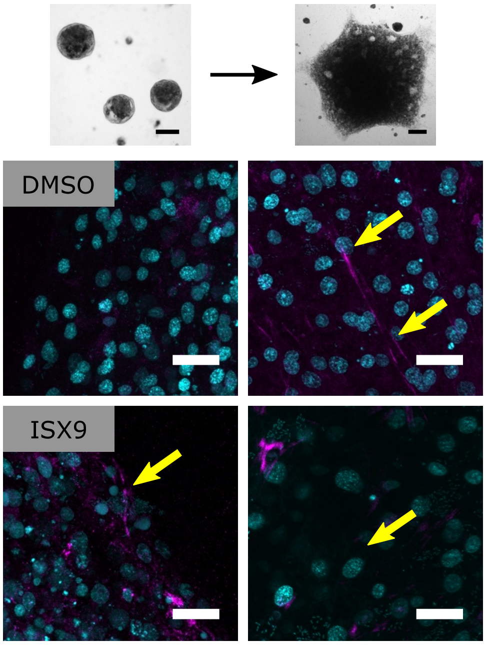

Supplementary Figure 6. Primmorphs treated with ISX9 stain for pRLC, suggesting a contractile phenotype. Top panel shows brightfield images of primmorphs (left) and a newly attached sponge (right). Middle panel shows pRLC staining (magenta) in a control, day 3 primmorph (left) and a new attached sponge (right). Signal along actin bundles (yellow arrows) becomes visible following attachment. Bottom panels shows a day 3 primmorph treated with ISX9 (left) and a treated sponge following attachment (right). pRLC signal is visible along linear structures in the treated primmorphs. DNA in cyan. Immunostaining of primmorphs was performed in two independent experiments with consistent results. Scale bars 100µm and 10µm.

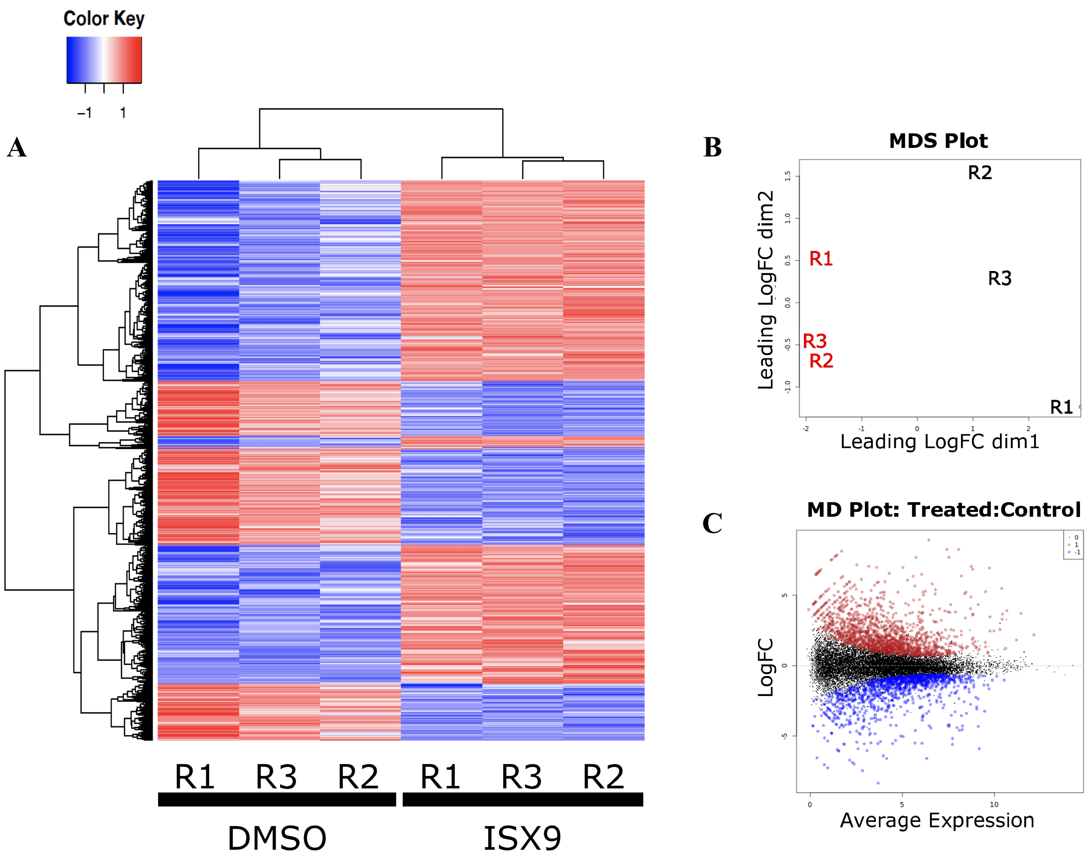

Supplementary Figure 7. Treatment shows a large and consistent transcriptional response in primmorphs. (A) Heatmap of full list of differentially expressed transcripts between treated and control sponges. Red shows upregulation in treated samples relative to control samples, while blue shows decreased expression. Rows and columns grouped based on clustering of normalized expression. **(B)** MDS plot for replicates of ISX9 treated (red) and DMSO (black) primmorphs. **(C)** MD plot for differentially expressed transcripts between treatment and control. Red shows significant increased expression in treatment and blue shows significantly decreased expression.

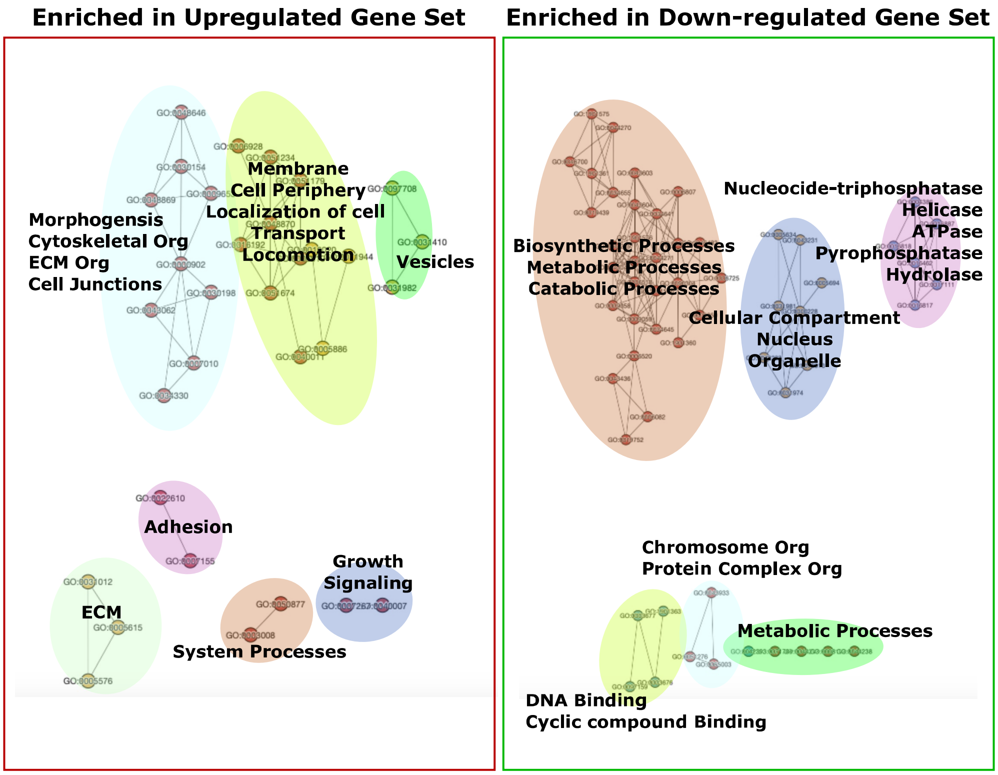

Supplementary Figure 8. GO-term analysis of differentially expressed transcripts. GO-term network visualized with NaviGO for GO-terms enriched in the upregulated gene set (red) and down-regulated gene set (green). Network clusters are colored to match supplementary table 1 and **2**. The Upregulated gene set contains transcripts involved in cell differentiation, cytoskeletal organization, and adhesion, while the down-regulated gene set contains transcripts involved in metabolic and catabolic processes, and DNA maintenance. GO-term analysis was performed using Blast2GO software[87]. A full list of terms enriched in the upregulated gene set is in supplementary table 1. A full list of terms enriched in down-regulated gene set is in supplementary table 2.

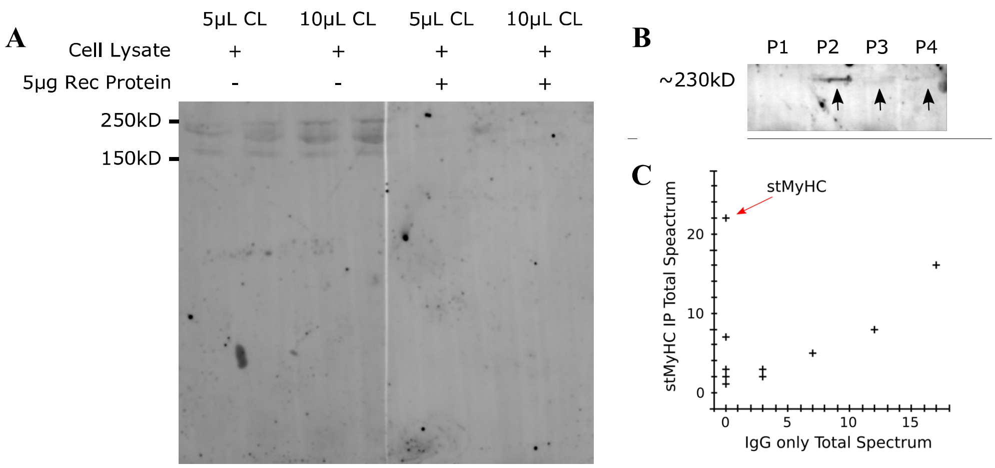

Supplementary Figure 9. Validation of custom EmstMyHC antibody. (A) Western blot of sponge whole cell lysates shows band at correct size for the predicted protein, which is lost following competition with recombinant peptide antigen. **(B)** Western blot of immunoprecipitation precipitates shows protein present in elutions 2-4. Competition blot was performed as a single experiment and immunoprecipitation with anti-stMyHC was performed three independent times with consistent results. **(C)** Results for mass spectrometry performed on stMyHC precipitates and IgG control precipitates show stMyHC as the strongest unique hit. Unadjusted and uncropped blots are shown in source data file.

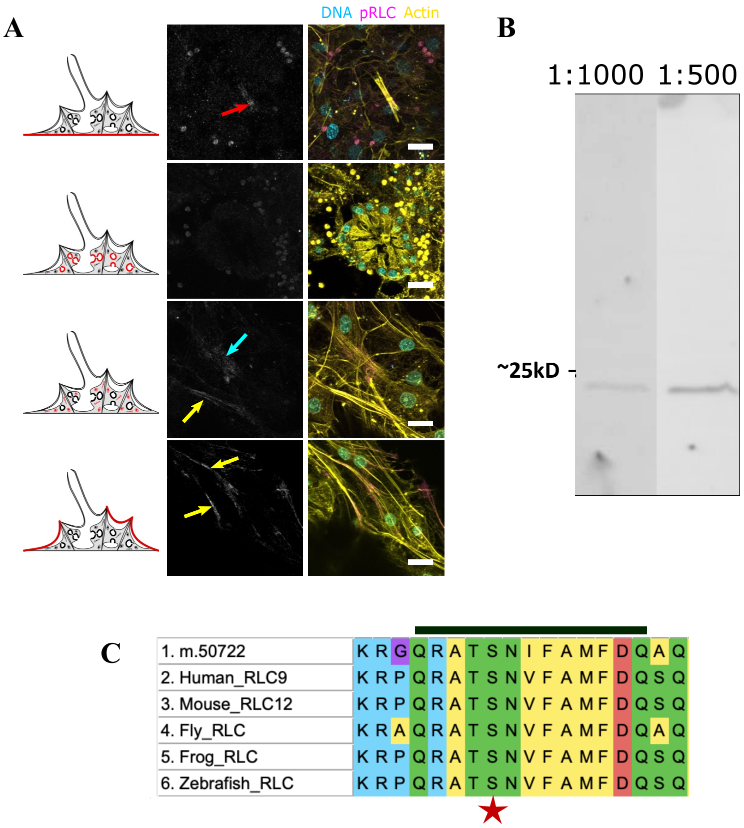

Supplementary Figure 10. Validation of pRLC antibody. (A) Confocal images of juvenile sponge Immunostained with pRLC (gray and magenta) counter stained with phalloidin (yellow) and hoechst (cyan). Tissue regions correspond with areas mark red in diagrams. Red arrow highlight signal at stress fiber-like structures in the attachment epithelia, yellow arrows highlight staining along actin bundles in tissue lining canals, and cyan arrow highlight diffuse signal in migratory cells in the mesohyl. **(B)** Western blot performed on whole cell lysates from juvenile sponges, blotted with 1:1000 (left) or 1:500 (right) dilution of primary antibody. pRLC antibody detects a single band approximately the predicted size of the protein. **(C)** Alignment of *E. muelleri* RLC (m.50722) with organisms listed by the manufacturer. The black bar indicates the maximum region the antibody was raised against, and the red star marks the phosphorylation site. Immunostainings for pRLC were performed on multiple sponges over three independent experiments with consistent patterns and western blot was performed on lysates from a single experiment. Scale bars 5µm. Unadjusted and uncropped blot is shown in source data file.

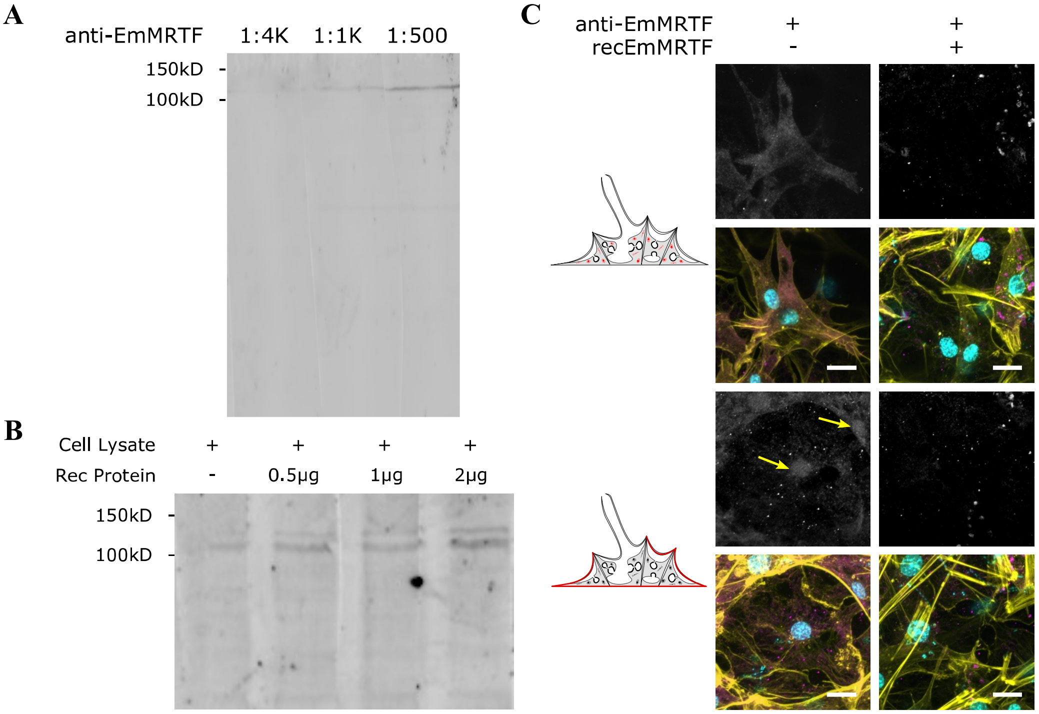

Supplementary Figure 11. Validation of custom EmMRTF antibody. (A) Western blot of sponge whole cell lysates using increasing concentrations of antibody shows a specific band at the predicted MW of the protein. **(B)** Western blot of sponge whole cell lysates blotted with anti-EmMRTF competed with increasing concentration of recombinant protein shows a loss in signal. **(C)** Immunofluorescent images of juvenile sponges stained with anti-EmMRTF either without recombinant protein (left) or with (right). Top portion shows migratory cells in the mesohyl and bottom portion shows pinacocytes. Grayscale images of raw antibody channel (top) and merged images show actin (yellow), EmMRTF (magenta), and DNA (cyan). Both broad cytoplasmic staining of migratory cells and nuclear staining (yellow arrows) of pinacocytes is lost following competition with recombinant protein. Western blots with anti-MRTF were performed in four independent experiments with consistent band pattern and. competitions were performed two independent times with consistent loss in signal. Scale bars 5μm. Unadjusted and uncropped blots are shown in source data file.

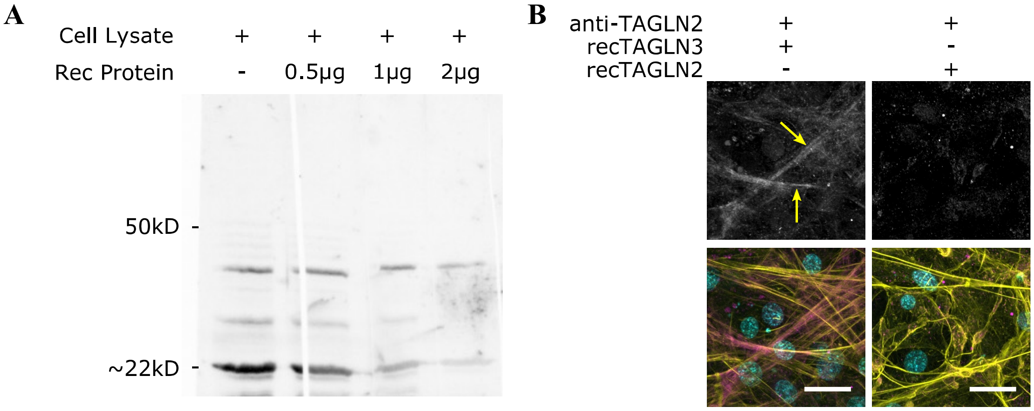

Supplementary Figure 12. Validation of custom EmTAGLN2 antibody. (A) Western blot of sponge whole cell lysates shows a band at the predicted size of the protein, which is lost with increasing concentration of recombinant protein. Higher MW bands are consistent with patterns seen for western blots of transgelins in other animals. **(B)** Immunofluorescent images of sponges stained with anti-EmTAGLN2 (magenta) pre-incubated with recombinant EmTAGLN3 (left) or recombinant EmTAGLN2 (right). Counter stained for actin (yellow) and DNA (cyan). Raw antibody channel (top) shows signal remains at actin bundles following competition with EmTAGLN3 (yellow arrows) but is lost following competition with EmTAGLN2. Competition blot was performed as a single experiment and competition immunostainings were performed in two independent experiments with consistent results. Scale bars 10 μm. Unadjusted and uncropped blot is shown in source data file.

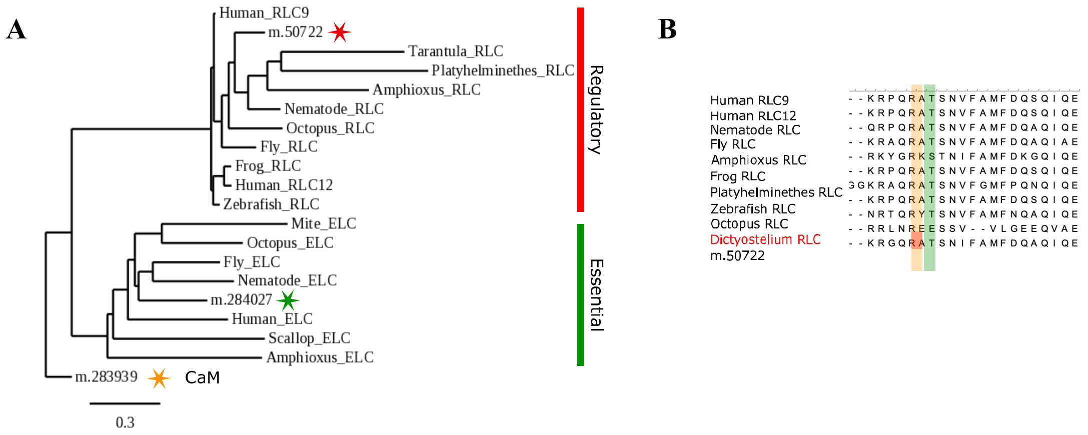

Supplementary Figure 13. *E. muelleri* orthologs of RLC and ELC. (A) Maximum likelihood phylogeny of evolutionarily related RLC and ELC rooted with the sponge EF-hand protein calmodulin (CaM). *E. muelleri* has an ortholog in the regulatory light chain group (red star) and the essential light chain group (green star). **(B)** Alignment of the region around the MLCK phosphorylation site showing conservation of phosphorylatable residues (highlighted in orange and green) and flanking regions in all animals including *E. muelleri* (m.50722).

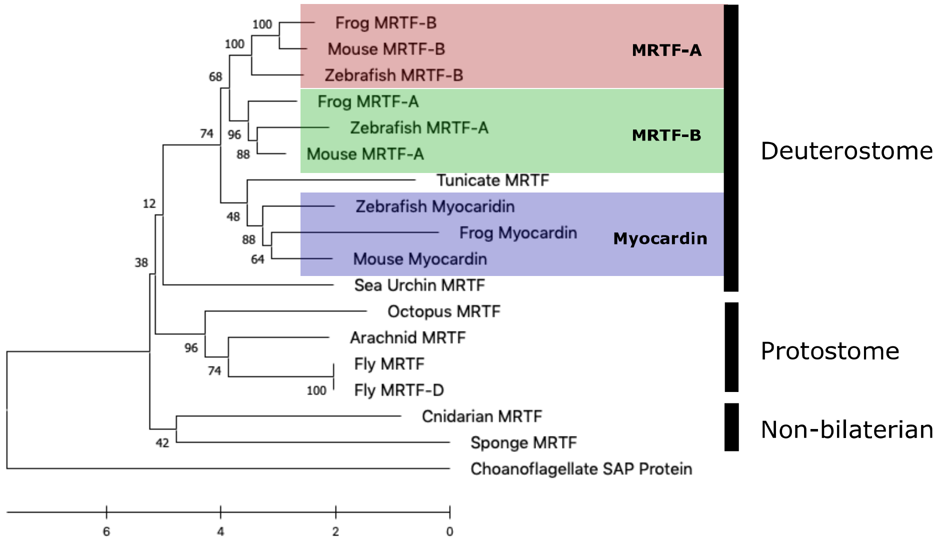

Supplementary Figure 14. MRTF family underwent expansion in vertebrate lineage. Maximum likelihood phylogeny of MRTF family proteins from a variety of animals, which closely matches their phylogenetic positioning. Single orthologs are generally present for non-bilaterians, protostomes, echinoderms, and hemichordates while vertebrate proteins can be divided into MRTF-A, MRTF-B, and myocardin. Choanoflagellate SAP domain containing protein was used as an outgroup. Support values based on 100 bootstrap iterations.

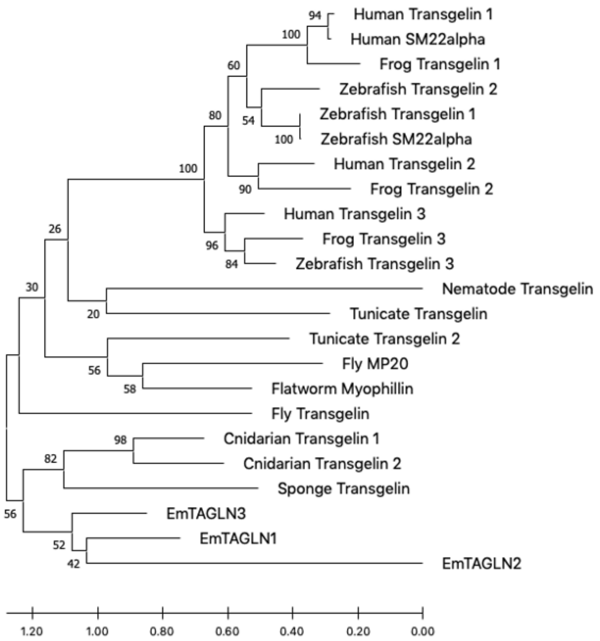

Supplementary Figure 15. ***E. muelleri*** **transgelins represent a lineage specific expansion**. Maximum likelihood phylogeny of transgelin family proteins from a variety of animals. Protein groupings correspond well to the species phylogeny, which suggests an expansion prior to the vertebrate radiation as well as in non- bilaterian lineages. Support values are based on 100 bootstrap iterations.

Protein and Primer Sequences Used in Study

Protein Sequences

Striated type II-Myosin heavy chain sequence from *E. muelleri*. Polyclonal antibody raised against peptide region highlighted in gray.

>m.68719 (stMyHC)
MTDSDPSVYLRPGRTTDITEQMRMFDAKKWLWLNDEEECFKAAFVKSQKGDKMVVELSNGS
EVTVDINATQQMNPPKFEKIEDMASLTYLNEASVLHNLRQRYYSSLIYTYSGLFCVAINPYRML
PVYTKTVIDMYRGKRKTEMPPHIFAVADNAYHDMLQDQENQSILITGESGAGKTENTKKVIQ
YFAVVAPSTQKQQTNLEDQVIQANPVLEAFGNAKTIRNDNSSRFGKFIRVHFGNQGKISGADIE
FYLLEKSRVIHQQAGERSYHIFYQIMAGASQDLLNKLLLKRQPKSYSFLANSELTVDNVDDSQ
MFKLTQEAMETLSFTEDEQMFLFKVIAGVLHFGNIEVKQRPREEWATIPTAEECDKVSHILGISS
ADLMKALIKPRIRVGNEYVQQGRNMDQVKYSIGALSKSLYERMFRWLVSRVNKTLDTKTRK
NFFIGVLDIAGFEIFKLNSFEQLCINYTNERLQQFFNHHMFVLEQEEYRKEGINWEFIDFGLDLQ
PCIDLIEKPLGIFSILDEECLFPKASDQSFINKLNANHADKSPNYIKAQFKTGGNTIDFEVAHYAG
TVGYTAAGWLDKNKDPLNDNVVELLKKSTDPGIASLWNDYLPEGERKGKGSQFVTMAHLHK
QSLNNLMTTLGNTTPHFVRCIIPNEMKRPGVIDAHLVLHQLRCNGVLEGIRICRKGFPNRLLYQ
EFRQRYIILAPKSIPAGFMDGRKATEALIEALQLENTEYRLGHSKVFFRAGVLGRLEDMRDERL
SIVLTQFQSFCRGYSTRKTYRKLLDQRLAIAVIQRNVRKHLFLRDWKWWKLYTKVKPLLNVA
RTEDELRQKEEELNKMREKLSKEEQGRKALEDIKTQLMEEKNQLFVQLQREHDACAEAEENL
QRLLGKKTDLEAHLQELVDRLDEEVENNANISAARRKVEAELEHHKESLTELKITLEQTVQEK
AQKEKDCATLDAELEKVNESLSRTNKEKTSLEERLQELTNSLQNEEDKANRLSKLKIKLESTIQ
ETHDELQKEKVARGELEKAKRKVEADLKSGQDALEQINRSKAELEKSLTGKDKEIADLVQKV
EEDQSQVAALQRRIRELEARIEELEEDLENERSQRQKAEKQKADLTKELETLQDQLEEEGGAK
TAQLELNKKREVELVQLRKDQERVSEDHEKVVADLRKKHAQSVGELEEKVDSLQKAKAKLE
KEKASLSTEGSDLVAQVQQMEKTKVATEKRLKQLEEQNGEISARLREYESTIATLQAAQAKA
QSENAELLQQATDAESKIGVITKAKNALEAQLDEAKAELESESNSKADIMVKLKAMESELNHA
HESIEEEQEGKTELQKQLTATKNDAAQLRARLDNEATPRIEELEDTKRKLQNKLKEAEEALTL
SDSKYAALDKTKTRIATELEDLHLDLEKERSNSAALENKQKKIDQQITEWRSKYESKQAELEN
AQKEARNYSTEVLKLKGQYDELHDQLEAANKENKHLQGEISDLEKQLSEGGSSVHDLEKAKR
KLEQEKEELTQSLEDAEGQLEGEQQRVLRIQLELTQLKQETERKLAEKDGEMESLRKNHGRQ
QESLQQSLEEESKAKNEQIRLKKVVEGQIDELQATIDAGEKAASDNQKKIKALQQQVKEITSSF
EEEQRLRSEDRDLTTKAEKRANDLQREIEELRVNMEQLQKSLRNIEAENADAQERLTEANNSK
TSLVASKRKVDQQLATLQGEYEELESEGKENSEKLRKATELVVRYQSEVMAEKEAANALEKA
RAALDQQVKDLTARLGEEVANATKSAKKEAQKLQARITELEGELETETKGKGDAQRNLKRL
DRRVKEVQAQMEEEKAASQRLQEQINSLNAKAKTLRREKEDTEGELEAMRTKNRQLRSALDE
SEEEKAGLQAQLSKARTTTTTSARKGKPAPVDADDDK

List of UniProt entries used to generate RLC/ELC tree
Fly ELC, sp|P54357; Human ELC, sp|P08590; Nematode ELC, sp|Q9XVI9; Scallop ELC, tr|Q26066; Mite ELC, tr|A0A6G1SHZ7; Amphioxus ELC, tr|A0A6P5AX12; Octopus, tr|A0A6P7SU30; Human RLC9, sp|P24844; Human RLC12, sp|O14950; Fly RLC, sp|P40423; Nematode RLC, sp|Q09510; Amphioxus RLC, tr|Q9NDR8; Frog RLC, tr|Q91625; Zebrafish RLC, tr|Q801M3; Octopus RLC, tr|A0A6P7T4N8; Platyhelminthes RLC, tr|A0A3R7GQV4

List of UniProt entries used to generate Transgelin tree
Human Transgelin 1, sp|Q01995; Human Transgelin 2, sp|P37802; Human Transgelin 3, sp|Q9UI15; Human SM22alpha, sp|Q5U0D2; Zebrafish Transgelin 1, tr|Q6P697; Zebrafish Transgelin 2, tr|Q6TGT5; Zebrafish Transgelin 3, tr|A2BEV4; Zebrafish SM22alpha, tr|Q29VH8; Frog Transgelin 1, tr|Q5U536; Frog Transgelin 2, tr|A0A1L8FCQ5; Frog Transgelin 3, tr|Q6P7J3; Fly Transgelin, tr|Q9VZI1; Nematode Transgelin, O44788; Fly MP20, sp|P14318; Tunicate Transgelin 2, tr|F7A7T2; Flatworm Myophillin, sp|Q24799; Tunicate Transgelin, tr|E4XQV3; Cnidarian Transgelin 1, tr|A7T059; Cnidarian Transgelin 2, tr|A7RPC0; Sponge Transgelin, tr|A0A1X7U007

***E. muelleri* transgelins sequences from transcriptome (Pena et al., 2016).**
EmTAGLN1, comp25280_c0_seq1; EmTAGLN2, comp47136_c0_seq2, EmTAGLN3, comp68850_c0_seq1

Predicted protein sequences. Polyclonal antibody raised against region highlighted in gray.

>EmTAGLN2_seq
MSTRAEKSGINAEVQQKLAATYSLEDEAKARGWLEAIVGESIGQDFWDGLKDGSYLCKVLNK
LQPNLVPAKYEHPNGQPFKQIEAVGKFLDGCKNYGMSEKDLCVTLDIQEKNNPNMGLATIIAL
GRQAQKNGYTGPALGPKEAQPCPHEIDEQRIREGRNVIGLQMGTNKGASQAGMTPYGHQRQI
NAP

>EmTAGLN3_seq
MADRPKGYGMTAEIQERLDAKYDAGLEAQAREWMELVVEEPFPEGSFQEALKDGTYLCKLM
NKLEPGAIRKINESKMAFKMMENIGLFLDACCAYGLTKVDLFQTVDLYDGTNIPQVINGIHAL
GRKAQVKGFSGAVLGPKEAAADKKEWTDEQLKAGQNIIGLQMGSNKGASAAGTTPFGLGRQ
IEKTNLKV

Sequences retrieved for MRTF tree:
Mouse MRTF-A, tr|E7F9J6; Zebrafish MRTF-A, sp|Q8K4J6; Frog MRTF-A, sp|Q8AYC2; Mouse MRTF-B, sp|P59759; Zebrafish MRTF-B, tr|U3JAU7; Frog MRTF-B, sp|Q8AYC1; Mouse myocardin, sp|Q8VIM5; Zebrafish myocardin, XP_005169482.1; Frog myocardin, tr|B0ZE99; Fly MRTF-D, tr|Q9VZY2; Fly MRTF, tr|Q6B4I4; Octopus MRTF, tr|A0A0L8H4W5; Arachnid MRTF, XP_015906589; Sea Urchin MRTF, SPU_005255.3a; Tunicate MRTF, jgi|Cioin2|287879; Choanoflagellate SAP protein, XP_001745005.1

Sequence for *E. muelleri* MRTF ortholog predicted from the transcriptome (Pena et al., 2016). Polyclonal antibody raised against region highlighted in gray.
>EmMRTF_Recombinant_Protein_Seq
MSSCEPLSLDWLDEELSASHSVDQRSMNRATVQGELEKRLRCRADRDDLVKKHILPDLSTSPV
LYGQTQLLQRAQIADTLKKKLSSRAERNELLQLNILPDLQTIIAPKLQAKQLELKKHKLADSLN
EKLANRPGPLELVREGILEPKNNTLAAMVQSVDGNNEPLSVEGGGGGHPTLSTCGFSDSEFLSP
PETQKISDSSSPTPSPREGSVEASSPVKSLMSPPTFPGSFSAMAAGAQQHFAHSMELGTRLGKA
MSPSVIRKKQQKQQKYRKLRYHEYIPPSKNNGKGGKTNQKTHTSSKPESPYSVLLQQQQLFLQ
LQVLQQQYPNGVLMQKLPDILKGIKQDGSSSSGGRKDSSGTGKSQTSPSGAEKSGSSSTPGLPQ
TVQVEQPNHLNATTIRFDELKVSDLKTACKEMRLIVSGKKAELVERLLEHNNGYLPVCALPDG
QSKDARKCSGAQSTASFDSSQASAASPMSPTLSSPIFKFPHIGGVSSSSVVDCLSSISPLGGPAMA
QVFPASNFQQQFDEIVERQKRSYISQKAPKTIAPRPELNDMVAIRFPCLEQRGVRGVGAPNNGG
RDGRCPPGGVQASKSLPTSPKNASPTDSAQSLLNELMESGEGVAGEDVKPPGFGGETLTSQPSG
LQSTRFSESLGLTTPSLSVMHSPSPHSVYIHQQQIHHHQQQQQQQQSQQRLHRASMPAVPGQN
PLQTLRYEQPLIQRSLSVAGVPLSQLSTSTSSSTTSYPSLPEMGSGGTGDLGKAMSSNGLLLGQD
EGLSMAVGGELMEIETSEFKDQPLSDILDIFPPGSNEYNAHQPHTLGYDNKALRVDMSSSDDIM
LSSHPLQPFGNSLNHQSRSTSDLCLRDNTKSNLGGGGGGYGNNHMISSEFGWLDLTLDNMGTF
GLSPSPSIGSAAAMSHSNPNSLMQDSNYMYFMDDSSVHSKGSNPPTGVSVHHHDATTFLEPVL
NPASHFFPTSEEATLLELGLSTS

Primer Sequences

Primer Sequences
Cloning Primers
EmMRTF
Forward – TACTTCCAATCCAATGCATCTTGCGAGCCGCTGAGCTT
Reverse – TTATCCACTTCCAATGTTATTACTAGCATGCAACATGAGTTA
EmTAGLN2
Forward – TACTTCCAATCCAATGCAGAAGTGCAGCAAAAGCTAGCAG
Reverse – TTATCCACTTCCAATGTTATTAGACCACAAGGCATGCACTAA
EmTAGLN3
Forward – TACTTCCAATCCAATGCAAAGGGATATGGCATGACTGC
Reverse – TTATCCACTTCCAATGTTATTAAGGTCCGTGAGATGCCTAA

qPCR Primers;
EmEF1alpha
Forward - AAGCCTATGTGCGTGGAGTCATTT
Reverse - GACACCTACTGCTACGGTCTGCTTC
EmGAPDH
Forward - GGTGATGGGTGTGAACGAAGA
Reverse - CAAGCAGTTGGTAGTGCAAGAGG
EmHptr1
Forward - ACGAATTTGTGGTGGGCTAC
Reverse - CACCACACGTACAGCTCGAT
EmActin
Forward - GACAATGGTTCAGGCATGTG
Reverse - TGGGATACTTCAGGGTGAGG
EmTAGLN2
Forward - AACCAACAAGGGTGCATCTC
Reverse - GACCACAAGGCATGCACTAA

Supplementary Table 1. GO terms enriched in up-regulated gene set

GO ID	GO Name	GO Category	FDR	P-Value
GO:0009653	anatomical structure morphogenesis	BIOLOGICAL_PROCESS	1.66E-05	1.79E-06
GO:0034330	cell junction organization	BIOLOGICAL_PROCESS	1.66E-05	1.81E-06
GO:0000902	cell morphogenesis	BIOLOGICAL_PROCESS	4.24E-05	4.80E-06
GO:0030198	extracellular matrix organization	BIOLOGICAL_PROCESS	1.36E-04	1.73E-05
GO:0043062	extracellular structure organization	BIOLOGICAL_PROCESS	1.36E-04	1.73E-05
GO:0048646	anatomical structure formation involved in morphogenesis	BIOLOGICAL_PROCESS	4.03E-04	6.02E-05
GO:0030154	cell differentiation	BIOLOGICAL_PROCESS	2.46E-03	4.12E-04
GO:0048869	cellular developmental process	BIOLOGICAL_PROCESS	2.46E-03	4.12E-04
GO:0007010	cytoskeleton organization	BIOLOGICAL_PROCESS	8.37E-03	2.23E-03
GO:0040011	locomotion	BIOLOGICAL_PROCESS	4.96E-09	6.82E-11
GO:0051179	localization	BIOLOGICAL_PROCESS	4.13E-08	1.65E-09
GO:0051674	localization of cell	BIOLOGICAL_PROCESS	4.69E-06	3.76E-07
GO:0006810	transport	BIOLOGICAL_PROCESS	4.69E-06	3.26E-07
GO:0006928	movement of cell or subcellular component	BIOLOGICAL_PROCESS	4.69E-06	3.76E-07
GO:0048870	cell motility	BIOLOGICAL_PROCESS	4.69E-06	3.76E-07
GO:0051234	establishment of localization	BIOLOGICAL_PROCESS	4.69E-06	3.26E-07
GO:0016192	vesicle-mediated transport	BIOLOGICAL_PROCESS	1.58E-05	1.62E-06
GO:0005886	plasma membrane	CELLULAR_COMPONENT	5.29E-09	1.54E-10
GO:0016020	membrane	CELLULAR_COMPONENT	5.29E-09	1.54E-10
GO:0071944	cell periphery	CELLULAR_COMPONENT	1.67E-08	5.48E-10
GO:0008092	cytoskeletal protein binding	MOLECULAR_FUNCTION	3.98E-03	6.82E-04
GO:0097708	intracellular vesicle	CELLULAR_COMPONENT	2.38E-04	3.44E-05
GO:0031982	vesicle	CELLULAR_COMPONENT	2.38E-04	3.44E-05
GO:0031410	cytoplasmic vesicle	CELLULAR_COMPONENT	2.38E-04	3.44E-05
GO:0022610	biological adhesion	BIOLOGICAL_PROCESS	5.98E-08	2.84E-09
GO:0007155	cell adhesion	BIOLOGICAL_PROCESS	5.98E-08	2.84E-09
GO:0005576	extracellular region	CELLULAR_COMPONENT	1.14E-11	4.15E-14
GO:0031012	extracellular matrix	CELLULAR_COMPONENT	8.14E-08	4.16E-09
GO:0005615	extracellular space	CELLULAR_COMPONENT	2.27E-02	7.28E-03
GO:0050877	nervous system process	BIOLOGICAL_PROCESS	4.99E-03	9.47E-04
GO:0003008	system process	BIOLOGICAL_PROCESS	6.91E-03	1.51E-03
GO:0040007	growth	BIOLOGICAL_PROCESS	8.37E-03	1.95E-03
GO:0007267	cell-cell signaling	BIOLOGICAL_PROCESS	2.75E-02	8.92E-03

Supplementary Table 2. GO terms enriched in down-regulated gene set

GO ID	GO Name	GO Category	FDR	P-Value
GO:0006725	cellular aromatic compound metabolic process	BIOLOGICAL_PROCESS	4.96E-09	1.09E-10
GO:1901360	organic cyclic compound metabolic process	BIOLOGICAL_PROCESS	4.96E-09	1.09E-10
GO:0046483	heterocycle metabolic process	BIOLOGICAL_PROCESS	4.96E-09	1.09E-10
GO:0006139	nucleobase-containing compound metabolic process	BIOLOGICAL_PROCESS	4.96E-09	1.09E-10
GO:0090304	nucleic acid metabolic process	BIOLOGICAL_PROCESS	4.13E-08	1.66E-09
GO:0006259	DNA metabolic process	BIOLOGICAL_PROCESS	5.90E-07	3.45E-08
GO:0009058	biosynthetic process	BIOLOGICAL_PROCESS	7.72E-06	6.76E-07
GO:0034641	cellular nitrogen compound metabolic process	BIOLOGICAL_PROCESS	8.19E-05	9.86E-06
GO:0006807	nitrogen compound metabolic process	BIOLOGICAL_PROCESS	6.82E-03	1.44E-03
GO:0006518	peptide metabolic process	BIOLOGICAL_PROCESS	8.37E-03	2.22E-03
GO:0006412	translation	BIOLOGICAL_PROCESS	8.37E-03	2.22E-03
GO:1901566	organonitrogen compound biosynthetic process	BIOLOGICAL_PROCESS	8.37E-03	2.22E-03
GO:0034645	cellular macromolecule biosynthetic process	BIOLOGICAL_PROCESS	8.37E-03	2.22E-03
GO:1901576	organic substance biosynthetic process	BIOLOGICAL_PROCESS	8.37E-03	2.22E-03
GO:0044249	cellular biosynthetic process	BIOLOGICAL_PROCESS	8.37E-03	2.22E-03
GO:0043043	peptide biosynthetic process	BIOLOGICAL_PROCESS	8.37E-03	2.22E-03
GO:0043604	amide biosynthetic process	BIOLOGICAL_PROCESS	8.37E-03	2.22E-03
GO:0043603	cellular amide metabolic process	BIOLOGICAL_PROCESS	8.37E-03	2.22E-03
GO:0009059	macromolecule biosynthetic process	BIOLOGICAL_PROCESS	8.37E-03	2.22E-03
GO:0044271	cellular nitrogen compound biosynthetic process	BIOLOGICAL_PROCESS	8.37E-03	2.22E-03
GO:1901361	organic cyclic compound catabolic process	BIOLOGICAL_PROCESS	1.44E-02	4.58E-03
GO:0006082	organic acid metabolic process	BIOLOGICAL_PROCESS	1.44E-02	4.43E-03
GO:0006520	cellular amino acid metabolic process	BIOLOGICAL_PROCESS	1.44E-02	4.43E-03
GO:0046700	heterocycle catabolic process	BIOLOGICAL_PROCESS	1.44E-02	4.58E-03
GO:0043436	oxoacid metabolic process	BIOLOGICAL_PROCESS	1.44E-02	4.43E-03
GO:0034655	nucleobase-containing compound catabolic process	BIOLOGICAL_PROCESS	1.44E-02	4.58E-03
GO:1901575	organic substance catabolic process	BIOLOGICAL_PROCESS	1.44E-02	4.58E-03
GO:0019752	carboxylic acid metabolic process	BIOLOGICAL_PROCESS	1.44E-02	4.43E-03
GO:0019439	aromatic compound catabolic process	BIOLOGICAL_PROCESS	1.44E-02	4.58E-03
GO:0044270	cellular nitrogen compound catabolic process	BIOLOGICAL_PROCESS	1.44E-02	4.58E-03
GO:0000228	nuclear chromosome	CELLULAR_COMPONENT	4.43E-07	2.43E-08
GO:0005694	chromosome	CELLULAR_COMPONENT	4.80E-06	4.03E-07
GO:0043233	organelle lumen	CELLULAR_COMPONENT	6.82E-03	1.47E-03
GO:0070013	intracellular organelle lumen	CELLULAR_COMPONENT	6.82E-03	1.47E-03
GO:0031981	nuclear lumen	CELLULAR_COMPONENT	6.82E-03	1.47E-03
GO:0031974	membrane-enclosed lumen	CELLULAR_COMPONENT	6.82E-03	1.47E-03
GO:0005634	nucleus	CELLULAR_COMPONENT	1.39E-02	3.75E-03
GO:0043231	intracellular membrane-bounded organelle	CELLULAR_COMPONENT	1.44E-02	4.28E-03
GO:0004386	helicase activity	MOLECULAR_FUNCTION	3.52E-06	2.19E-07
GO:0016887	ATPase activity	MOLECULAR_FUNCTION	1.88E-04	2.47E-05
GO:0016462	pyrophosphatase activity	MOLECULAR_FUNCTION	4.01E-03	7.46E-04
GO:0016817	hydrolase activity, acting on acid anhydrides	MOLECULAR_FUNCTION	4.01E-03	7.46E-04
GO:0016818	hydrolase activity, acting on acid anhydrides, in phosphorus-containing anhydrides	MOLECULAR_FUNCTION	4.01E-03	7.46E-04
GO:0017111	nucleoside-triphosphatase activity	MOLECULAR_FUNCTION	4.01E-03	7.46E-04
GO:1901363	heterocyclic compound binding	MOLECULAR_FUNCTION	8.96E-06	8.83E-07
GO:0003676	nucleic acid binding	MOLECULAR_FUNCTION	8.96E-06	8.83E-07
GO:0097159	organic cyclic compound binding	MOLECULAR_FUNCTION	8.96E-06	8.83E-07
GO:0003677	DNA binding	MOLECULAR_FUNCTION	2.38E-04	3.47E-05
GO:0051276	chromosome organization	BIOLOGICAL_PROCESS	7.16E-05	8.36E-06
GO:0065003	protein-containing complex assembly	BIOLOGICAL_PROCESS	1.44E-02	4.12E-03
GO:0043933	protein-containing complex subunit organization	BIOLOGICAL_PROCESS	1.44E-02	4.12E-03
GO:0044237	cellular metabolic process	BIOLOGICAL_PROCESS	4.52E-04	6.92E-05
GO:0008152	metabolic process	BIOLOGICAL_PROCESS	1.01E-03	1.58E-04
GO:0071704	organic substance metabolic process	BIOLOGICAL_PROCESS	5.05E-03	9.95E-04
GO:0044238	primary metabolic process	BIOLOGICAL_PROCESS	5.05E-03	9.95E-04
GO:0042393	histone binding	MOLECULAR_FUNCTION	2.46E-03	3.99E-04
