# Supplementary material for: MRTF specifies a muscle-like contractile module in Porifera
Source: Nat Commun. 2022 Jul 15;13:4134. doi: 10.1038/s41467-022-31756-9 (PMC9287330; doi:10.1038/s41467-022-31756-9)
Supplement: Supplementary file 2 — Description of Additional Supplementary Files [file 41467_2022_31756_MOESM2_ESM.pdf]

Description of Additional Supplementary Files

File Name: Supplementary Movie 1
Description: Time lapse of *E. muelleri* mechanical contraction

File Name: Supplementary Movie 2
Description: Time lapse of *E. muelleri* thapsigargin contraction

File Name: Supplementary Movie 3
Description: Time lapse of *E. muelleri* L-NAME treated with ink

File Name: Supplementary Movie 4
Description: Time lapse of *E. muelleri* L-NAME treated with thapsigargin

File Name: Supplementary Movie 5
Description: Time lapse of *E. muelleri* ML-7 treated with thapsigargin

File Name: Supplementary Data 1
Description: Full list of differentially expressed transcripts
