# Supplementary material for: MRTF specifies a muscle-like contractile module in Porifera
Source: Nat Commun. 2022 Jul 15;13:4134. doi: 10.1038/s41467-022-31756-9 (PMC9287330; doi:10.1038/s41467-022-31756-9)
Supplement: Supplementary file 9 — Reporting Summary [file 41467_2022_31756_MOESM9_ESM.pdf]

nature portfolio

Corresponding author(s): Nichols, Scott A

Last updated by author(s): 2022/06/08

Reporting Summary

Nature Portfolio wishes to improve the reproducibility of the work that we publish. This form provides structure for consistency and transparency in reporting. For further information on Nature Portfolio policies, see our Editorial Policies and the Editorial Policy Checklist.

Statistics

For all statistical analyses, confirm that the following items are present in the figure legend, table legend, main text, or Methods section.

n/a	Confirmed	
☐	☒	The exact sample size (*n*) for each experimental group/condition, given as a discrete number and unit of measurement
☐	☒	A statement on whether measurements were taken from distinct samples or whether the same sample was measured repeatedly
☐	☒	The statistical test(s) used AND whether they are one- or two-sided<br>*Only common tests should be described solely by name; describe more complex techniques in the Methods section.*
☐	☒	A description of all covariates tested
☐	☒	A description of any assumptions or corrections, such as tests of normality and adjustment for multiple comparisons
☐	☒	A full description of the statistical parameters including central tendency (e.g. means) or other basic estimates (e.g. regression coefficient) AND variation (e.g. standard deviation) or associated estimates of uncertainty (e.g. confidence intervals)
☐	☒	For null hypothesis testing, the test statistic (e.g. *F*, *t*, *r*) with confidence intervals, effect sizes, degrees of freedom and *P* value noted<br>*Give P values as exact values whenever suitable.*
☒	☐	For Bayesian analysis, information on the choice of priors and Markov chain Monte Carlo settings
☒	☐	For hierarchical and complex designs, identification of the appropriate level for tests and full reporting of outcomes
☒	☐	Estimates of effect sizes (e.g. Cohen's *d*, Pearson's *r*), indicating how they were calculated

Our web collection on statistics for biologists contains articles on many of the points above.

Software and code

Policy information about availability of computer code

Data collection	Time-lapse images were acquired using Qimaging Q-capture Pro7 software (version 7.0.427.30). Confocal images were acquired using Olympus Fluoview FV315-SW software. qPCR data was acquired using BioRad iQ5 Optical System Software (version 2.1).
Data analysis	Image analysis was perfored using Fiji (Version 2.0.0-rc-69/1.52p). Statistics were performed using Rstudio Desktop (version 0.98.507), JMP Version 15 software (SAS Institute Inc., Cary, NC), and Microsoft Excel (version 16). RNAseq analysis was performed using fastp (version 0.19.5), hisat2 (version 2.1.0), StringTie (version 2.1.1), htseq-count (version 0.9.1), EdgeR (version 3.34.0), and BLAST2GO Pro (version 1.3.11). Analysis of protein homology was performed using HMMER (version 3.3.2) and MABL phylogeny.fr (version 1.0).

For manuscripts utilizing custom algorithms or software that are central to the research but not yet described in published literature, software must be made available to editors and reviewers. We strongly encourage code deposition in a community repository (e.g. GitHub). See the Nature Portfolio guidelines for submitting code & software for further information.

Data

Policy information about availability of data

All manuscripts must include a data availability statement. This statement should provide the following information, where applicable:

- Accession codes, unique identifiers, or web links for publicly available datasets
- A description of any restrictions on data availability
- For clinical datasets or third party data, please ensure that the statement adheres to our policy

Sequences and accession numbers for all genes and proteins analyzed in this study are made available in the supplement. Raw RNAseq reads have been deposited at the NCBI SRA (accession number/BioProject PRJNA718521, BioSamples SAMN18537458 [https://www.ncbi.nlm.nih.gov/sra/SRX10474595[accn]], SAMN18537459 [https://www.ncbi.nlm.nih.gov/sra/SRX10474596[accn]], SAMN18537460 [https://www.ncbi.nlm.nih.gov/sra/SRX10474591[accn]],

SAMN18537461 [https://www.ncbi.nlm.nih.gov/sra/SRX10474592[accn]], SAMN18537462 [https://www.ncbi.nlm.nih.gov/sra/SRX10474593[accn]], SAMN18537463 [https://www.ncbi.nlm.nih.gov/sra/SRX10474594[accn]]). Raw confocal image files are available at figshare (#######).

Field-specific reporting

Please select the one below that is the best fit for your research. If you are not sure, read the appropriate sections before making your selection.

☒ Life sciences ☐ Behavioural & social sciences ☐ Ecological, evolutionary & environmental sciences

For a reference copy of the document with all sections, see nature.com/documents/nr-reporting-summary-flat.pdf

Life sciences study design

All studies must disclose on these points even when the disclosure is negative.

Sample size

No sample size calculations were performed. Sample size was chosen based on practicality for the system, based on factors such as length of time the contractile behavior takes, how long juvenile sponges are viable in the lab, and culturing conditions. Assays to allow higher throughput screening were designed in order to maximize the sampling size for this system.

Data exclusions

No data was excluded from the analysis

Replication

All immunostainings were performed on multiple individual sponges with secondary-only controls performed in each experiment for establishing detection levels. General tissue staining of stMyHC was performed in three independent experiments with consistent results. Developmental series staining for stMyHC and actin was performed in two independent experiments with consistent results. Latrunculin-B treatment was performed in two independent experiments with consistent results. Immunostaining for pRLC was performed in three independent experiments with consistent results. Immunostaining for general MRTF distribution was performed in five independent experiments. Results showed consistent patterns with some variation in signal intensity relative to background autofluorescence. TAGLN2 immunostainings were performed in four independent experiments with consistent results and competition stainings (with recombinant TAGLN2 and TAGLN 3) were performed in two independent experiments with consistent results. Unattached primmorphs treated with ISX were stained for actin in two independent experiments with consistent results and were also stained for pRLC in two independent experiments, with consistent results, though counterstaining for actin failed. Contraction videos were all performed >three times with full analysis performed on independent samples for each treatment shown, with consistent results. Mechanical agitation does not reliably induce contraction, but when it does, canal dynamics are consistent. Ink, thapsigargin, and ionomycin consistently induced contractions in independent experiments. Contraction assays (shown in Fig4D and Fig5E) were optimized over several treatments to identify effective concentration. Following this, they were performed in three independent experiments with consistent results. qPRC experiments were performed on samples from three independent experiments with consistent results. Primmorphs were treated with ISX and maintained in an attachment free environment 9 independent times with consistent morphologies. Of these, total protein extraction was performed on one sample, and total RNA was extracted from three independent experiments with consistent results. Treatment of sponges with PAB following treatment with thapsigargin was repeated in independent experiments with consistent results. Immunoprecipitation with anti-stMyHC was performed in three independent experiments, with consistent results and mass spectrometry performed on the final sample.

Randomization

Gemmules collected from the same adult sponge are genetically clonal. Controls were always taken from the same gemmule stock as the experimental group to insure assays were performed in the same genetic background.

Blinding

Blinding was not possible due to COVID-related personnel restrictions at the time experiments were conducted. The majority of data analyzed involved quantitative measurements and are not susceptible to biases. When possible, image analysis was performed in bulk so investigators did not observe trends until organizing the results tables.

Reporting for specific materials, systems and methods

We require information from authors about some types of materials, experimental systems and methods used in many studies. Here, indicate whether each material, system or method listed is relevant to your study. If you are not sure if a list item applies to your research, read the appropriate section before selecting a response.

Materials & experimental systems

n/a	Involved in the study	
☐	☒	Antibodies
☒	☐	Eukaryotic cell lines
☒	☐	Palaeontology and archaeology
☐	☒	Animals and other organisms
☒	☐	Human research participants
☒	☐	Clinical data
☒	☐	Dual use research of concern

Methods

n/a	Involved in the study	
☒	☐	ChIP-seq
☒	☐	Flow cytometry
☒	☐	MRI-based neuroimaging

Antibodies

Antibodies used	Anti-vinculin (Ephydatia muelleri); anti-stMyHC (Ephydatia muelleri, custom-this study); anti-MRTF (Ephydatia muelleri, custom-this study); anti-TAGLN2 (Ephydatia muelleri, custom-this study); anti-TALN3 (Ephydatia muelleri, custom-this study); anti-pRLC (Cell Signaling Technology #3671T); AlexaFluor® 488 Goat Anti-Rabbit IgG (Lifetechnologies); AlexaFluor® 647 Goat Anti-Chicken IgY (Invitrogen #A32933).
Validation	Anti-vinculin was raised against recombinant protein cloned from cDNA of E. muelleri and was validated through immunoprecipitation followed by mass spec, described in Mitchell JM, Nichols SA. 2019. Diverse cell junctions with unique molecular composition in tissues of a sponge (Porifera). EvoDevo, 10; 26.; anti-stMyHC was custom raised for this study and validated with immunoprecipitation followed by mass spec as described in methods. Validations shown in supplement. anti-MRTF was custom raised against recombinant proteins cloned from cDNA of E. muelleri for this study. Validation was performed through competition with recombinant protein during western blot and immunostaining. Attempts to use it in immunoprecipitation were unsuccessful. Validation steps are described in the methods and shown in supplement. anti-TAGLN2 was custom raised for this study against recombinant protein cloned from cDNA of E. muelleri. Validation was performed through competition with recombinant protein during western blot and immunostaining. Attempts to use it in immunoprecipitation were unsuccessful. Validation steps are described in the methods and shown in supplement. anti-TAGLN3 was custom raised for this study against recombinant protein cloned from cDNA of E. muelleri. Validation was performed through competition with recombinant protein during western blot and immunostaining. Attempts to use it in immunoprecipitation were unsuccessful. Validation steps are described in the methods and shown in supplement. anti-pRLC was raised against synthetic phosphopeptide corresponding to residues surrounding serine 19 of human myosin regulatory light chain and phospho-specificity was validated by manufacturer described at (https://www.cellsignal.com/products/primary-antibodies/phospho-myosin-light-chain-2-ser19-antibody/3671). Antibody was used in the choanoflagellate Choanoeca flexa in Brunet, T, Larson, B, Linden, T, Vermeij, M, McDonald, K, and King, N. 2019. Light-regulated collective contractility in a multicellular choanoflagellate. Science. 366(6463) p.326-334. The myosin regulatory light chain ortholog in E. muelleri shows a similarly high degree of conservation at the antigen region. Antibody was validated for E. muelleri by western blot.

Animals and other organisms

Policy information about studies involving animals; ARRIVE guidelines recommended for reporting animal research

Laboratory animals	The study did not involve laboratory animals
Wild animals	The study did not involve wild animals
Field-collected samples	Sponge tissue containing gemmules was collected and returned to the laboratory in lakewater. Samples were transferred to autoclaved lakewater and stored at 4oC under continual darkness. Gemmules were separated from sponge tissue and washed with 1% hydrogen peroxide prior to hatching. Hatching and development into juvenile sponges was performed by placing gemmules in autoclaved lakewater and maintaining at room temperature in the dark. Following experiments, all animals were disposed of.
Ethics oversight	No ethical approval or guidance was required for work with invertebrate animals.

Note that full information on the approval of the study protocol must also be provided in the manuscript.
